# Methods for the Comprehensive *in vivo* Analysis of Energy Flux, Fluid Homeostasis, Blood Pressure, and Ventilatory Function in Rodents

**DOI:** 10.3389/fphys.2022.855054

**Published:** 2022-02-25

**Authors:** John J. Reho, Pablo Nakagawa, Gary C. Mouradian, Connie C. Grobe, Fatima L. Saravia, Colin M. L. Burnett, Anne E. Kwitek, John R. Kirby, Jeffrey L. Segar, Matthew R. Hodges, Curt D. Sigmund, Justin L. Grobe

**Affiliations:** ^1^Department of Physiology, Medical College of Wisconsin, Milwaukee, WI, United States; ^2^Comprehensive Rodent Metabolic Phenotyping Core, Medical College of Wisconsin, Milwaukee, WI, United States; ^3^Cardiovascular Center, Medical College of Wisconsin, Milwaukee, WI, United States; ^4^Neuroscience Research Center, Medical College of Wisconsin, Milwaukee, WI, United States; ^5^Department of Pediatrics, Medical College of Wisconsin, Milwaukee, WI, United States; ^6^Department of Microbiology and Immunology, Medical College of Wisconsin, Milwaukee, WI, United States; ^7^Department of Internal Medicine, University of Iowa Hospitals & Clinics, Iowa City, IA, United States; ^8^Department of Biomedical Engineering, Medical College of Wisconsin, Milwaukee, WI, United States

**Keywords:** cardiovascular, metabolic, ventilatory, blood pressure, phenotyping, microbiome, mouse, rat

## Abstract

Cardiovascular disease represents the leading cause of death in the United States, and metabolic diseases such as obesity represent the primary impediment to improving cardiovascular health. Rodent (mouse and rat) models are widely used to model cardiometabolic disease, and as a result, there is increasing interest in the development of accurate and precise methodologies with sufficiently high resolution to dissect mechanisms controlling cardiometabolic physiology in these small organisms. Further, there is great utility in the development of centralized core facilities furnished with high-throughput equipment configurations and staffed with professional content experts to guide investigators and ensure the rigor and reproducibility of experimental endeavors. Here, we outline the array of specialized equipment and approaches that are employed within the Comprehensive Rodent Metabolic Phenotyping Core (CRMPC) and our collaborating laboratories within the Departments of Physiology, Pediatrics, Microbiology & Immunology, and Biomedical Engineering at the Medical College of Wisconsin (MCW), for the detailed mechanistic dissection of cardiometabolic function in mice and rats. We highlight selected methods for the analysis of body composition and fluid compartmentalization, electrolyte accumulation and flux, energy accumulation and flux, physical activity, ingestive behaviors, ventilatory function, blood pressure, heart rate, autonomic function, and assessment and manipulation of the gut microbiota. Further, we include discussion of the advantages and disadvantages of these approaches for their use with rodent models, and considerations for experimental designs using these methods.

## Introduction

Metabolic diseases characterized by increased adiposity affect >30% of the global population and have become a significant public health crisis in the past several decades (Roger et al., 2020). In parallel, hypertension is present in >30% of the global population, and there is gross overlap between these two groups (Virani et al., 2021). While cardiovascular disease remains the primary cause of death in the United States, the American Heart Association has indicated that obesity, along with hypertension, represent the primary impediments to ongoing improvements in cardiovascular health (Roger et al., 2020; Angell et al., 2020).

Obesity is a complex disease of dysregulated energy balance resulting from genetic, environmental, and behavioral causes. It has been estimated that at the population level, human obesity is a result of a sustained ≈7 kcal/d imbalance (equating to an ≈0.35% imbalance in a typical 2,000 kcal/d turnover) (Hall et al., 2011). Obesity resulting from such a small chronic imbalance illustrates the efficiency of the body, the critical importance of each input and output mechanism in governing energy balance, and the potential role of each mechanism as both a cause and therapeutic target for obesity. It follows that careful, rigorous, reproducible, integrative, and simultaneous study of all the mechanisms of energy flux is required to fully understand the impact of experimental interventions.

**Figure 1:**
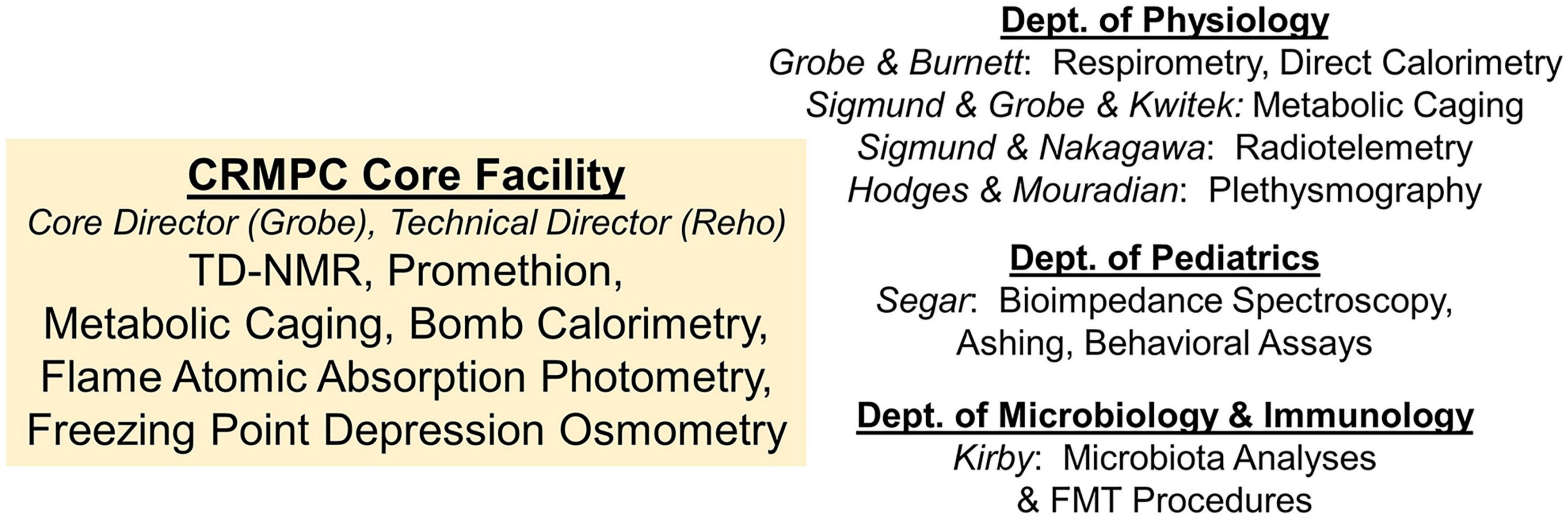
Overall structure of the MCW CRMPC and collaborating laboratories. Equipment and technical expertise provided by our collaborative team is provided both directly through the CRMPC facility and through collaborating laboratories from departments across the institution.

In 2019, the Comprehensive Rodent Metabolic Phenotyping Core (CRMPC) was established at the Medical College of Wisconsin (MCW) to provide quantitative and comprehensive assessments of energy and fluid homeostasis in rodents, to assist in the dissection of complex and integrated mechanisms of metabolic disease. To accomplish this mission, the CRMPC provides investigators with easy, low-barrier, and guided access to advanced technologies and provides rigorously standardized measurements of energy flux and fluid homeostasis in rodents. Simultaneously, through our research laboratories across various departments within MCW, our team is also well positioned to provide parallel experimental support of studies investigating cardiovascular, metabolic, ventilatory, and autonomic functions, in addition to studies and manipulations of the gut microbiota (Figure 1). Significantly, the CRMPC has developed technologies and workflows to support studies in both mouse and rat models, to leverage both the outstanding availability of genetically modified mouse models and to support the increasing availability of genetically modified rat models available for such investigations (Kwitek, 2018). In addition, the design and engineering of our equipment for both species is time and cost efficient and reduces our overall space requirements. Although the focus of discussion throughout this manuscript is upon the use of this type of instrumentation for use with mice and rats, it should be noted that many or all of the approaches can be applied to study of other similarly sized rodents, such as hamsters, guinea pigs, prairie dogs, and squirrels. Thus, through the development of a centralized core facility, equipped with cutting-edge technologies optimized for many species and staffed with appropriate content experts, we are positioned to maximize efficiency, minimize cost, reduce required animal numbers, refine methods and technologies for these types of studies, and ultimately work toward replacement of outdated and inefficient approaches.

A distinctive feature of the CRMPC is unique instrumentation to provide deep mechanistic dissection of resting metabolic rate (RMR) in rodents, including anaerobic aspects that very few metabolic phenotyping facilities are equipped to assess. Increasing evidence demonstrates that following the development of obesity, the human body resists the maintenance of weight loss primarily through adaptation (i.e., suppression) of RMR (Fothergill et al., 2016; Oliveira et al., 2021). Increasing evidence also supports a sizeable contribution of anaerobic metabolism to human and animal energy expenditure; that this flux is largely contributed by gut bacteria; and that this contribution is essentially lost during obesity (Pittet et al., 1974, 1976; Walsberg and Hoffman, 2005; Kaiyala and Ramsay, 2011; Burnett and Grobe, 2013, 2014; Bahr et al., 2015; Riedl et al., 2021). Therefore, the development of workflows to routinely and precisely assess the status of the gut microbiota, and the development and optimization of technologies to assess both aerobic and anaerobic metabolism *in vivo*, represent notable recent technological advancements of the CRMPC.

In this report, we aim to highlight workflows utilized by the CRMPC and collaborative laboratories at MCW for comprehensive cardiometabolic phenotyping in rodents. We also highlight considerations of the CRMPC with the aim to enhance rigor and reproducibility for cardiometabolic phenotyping in rodents and provide a discussion on the advantages and disadvantages of several commercially available metabolic phenotyping equipment designs currently on the market for investigators. Through the description of technologies and approaches immediately available within the CRMPC, the current manuscript will simultaneously serve as a technical guide for local facility users, an educational resource for investigators and trainees beginning projects to investigate cardiometabolic function in rodents, and as an advertisement to facilitate new ideas and collaborations.

## Generalized Overview of the CRMPC Workflow

**Figure 2:**
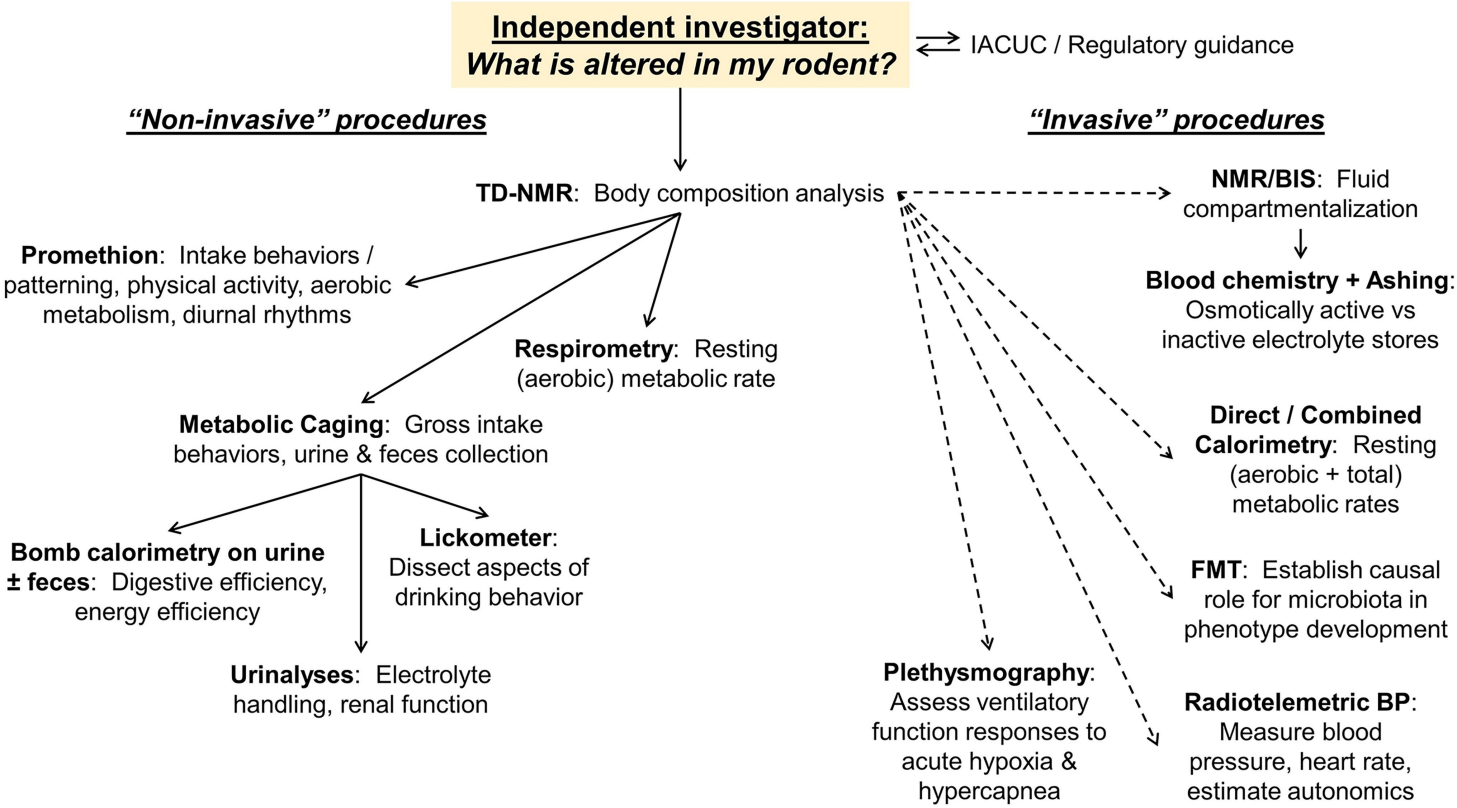
Workflow and endpoints available to users. Examples of both non-invasive and invasive endpoints (ranging from only mild temporary sedation through terminal procedures) available to end users, and the utility of these endpoints, are illustrated as a guide for discussions with new investigators approaching the core facility for phenotyping assistance.

A generalized workflow of the CRMPC and its collaborating laboratories is shown in Figure 2. In this example, we illustrate options for an independent investigator that approaches the CRMPC for assistance in understanding a body mass phenotype in their rodent model of interest.

First, after an initial consultation, the CRMPC provides guidance in study design and IACUC protocol development. Such guidance accelerates the process for new users and reduces administrative burdens for both the investigator and for IACUC administrative staff, as the CRMPC staff can provide template language and advice with regard to necessary considerations in protocol development.

To begin comprehensive phenotyping assessments of animals, an array of non-invasive procedures may be applied to rapidly generate a vast array of information about the cardiometabolic status of the animals (e.g., Figure 2, left side). Importantly, as they are non-invasive, these many procedures can be applied to the same animals, which reduces the overall number of animal subjects required for these analyses. Subsequently, multiple additional steps can be taken to more deeply dissect and investigate individual mechanisms.

Often, the first step will be to determine any changes in body composition. Is the animal model of interest large/small due to changes in specific mass compartments such as fat or fat-free compartments, or changes in hydration? To address such questions, the CRMPC utilizes time-domain nuclear magnetic resonance (NMR) to rapidly and non-invasively assess body composition. In addition, we may employ minimally invasive bioimpedance spectroscopy (BIS) in combination with NMR to quantify the relative distribution of fluids between extracellular and intracellular compartments.

Next, typically, we will assess ingestive behaviors and perform quantitative collections of urine and feces for other analyses using species-specific metabolic caging systems. Similar assessments of ingestive behaviors (but not urine or fecal production) can be performed in static home cages. To further explore nuanced aspects of ingestive behavior and taste psychophysics, we might also employ lickometers or brief-access methods.

After quantitative collection of urine and feces in metabolic caging systems, our team will often perform analysis of fecal and/or urine energy density *via* bomb calorimetry. The CRMPC utilizes high-resolution semi-micro bomb calorimetry to determine energy (calories) lost to feces and urine as well as caloric density of food, ultimately to determine the fraction of calories absorbed by the gut (i.e., digestive efficiency). Further, an integrated but inverse metric of whole-body energy expenditure (i.e., energy efficiency) can be calculated by determining the ratio of body mass gains over time per total caloric absorption during that same time period. Fluid/electrolyte homeostasis is also determined from data collected in metabolic cages using both freezing-point depression osmometry and flame atomic absorption spectroscopy as applied to drink and food sources, and urine and fecal samples.

To investigate the RMR of a rodent, O_2_/CO_2_ respirometry (one common form of “indirect” calorimetry) is also easily applied. While housed in a warmed testing chamber, gas exchange with the environment is monitored while the animal rests. Empirically derived formulae are then used to estimate heat production by the animal based on the rates of gas exchange (Grobe, 2017). The technique is especially useful when investigating the effects of acute pharmacological intervention in an animal model.

In another high-throughput, non-invasive approach provided by the CRMPC, animals can be studied in a multiplexed metabolic phenotyping system for extended periods of time. Multiple vendors offer different designs for such systems; currently, the CRMPC operates a 16-chamber rat-or-mouse Promethion system (Sable Systems International). This system allows for essentially continuous assessment of aerobic metabolism (VO_2_, VCO_2_, and RER), food and water intake, body weight, and physical locomotor activity across a full week of recording time. From these data, the investigator can assemble a 24 h track of major components of energy flux. Further, the investigator can dissect patterns of feeding, drinking, motion, and sleep to generate a full behavioral matrix in their animal model of interest.

Following these many non-invasive assessments of cardiometabolic function, several invasive technologies and techniques may be subsequently employed to investigate individual mechanisms. For example, after NMR/BIS assessments of body fluid compartmentalization, some studies may focus on electrolyte balance. Blood collection and analyses may therefore be appropriate. A handheld iSTAT clinical chemistry analyzer (Abbott) can rapidly provide a vast array of electrolyte and solute assessments from a very small volume of blood. Additionally, whole-body electrolytes may be assessed by using an ashing approach, in which the whole body is reduced to ash by stepwise baking (eventually up to 600°C) in a specialized oven, and electrolytes are then analyzed in the reconstituted ash *via* flame photometry.

As stated above, the CRMPC is uniquely instrumented to assess the emerging role of gut microbiota in the regulation of cardiometabolic phenotypes. We provide sterile fecal collection services which enable longitudinal studies of the effect of interventions upon the status of the gut microbiota. As an extension of these collections, we are subsequently positioned to perform fecal material transplant (FMT) studies, in which fecal material collected from “donor” cohorts of animals can be passed to “recipient” animals *via* gastric gavage (Bahr et al., 2015). Such FMT experiments represent a critical step in establishing causality of the gut microbiota in the generation of cardiometabolic phenotypes.

In another example, following assessments of energy expenditure (by bomb calorimetry, respirometry, and/or multiplexed system), gaps in energy balance may be apparent. For example, we have previously demonstrated that significant fractions of total energy intake can be “missing” after accounting for digestive efficiency, growth, and aerobic expenditure (Soto et al., 2019). Further, multiple studies now support both theoretically (Mansell and Macdonald, 1990; Kaiyala and Ramsay, 2011; Kaiyala, 2014) and empirically (Pittet et al., 1974, 1976; Walsberg and Hoffman, 2005; Burnett and Grobe, 2013, 2014; Soto et al., 2019) that O_2_/CO_2_ respirometry is blind to anaerobic metabolism in various species and that anaerobic metabolism contributes a physiologically and pathophysiologically significant fraction of total energy flux (Riedl et al., 2017, 2021). To assess anaerobic metabolism *in vivo*, our team has employed “combined calorimetry,” a technique that involves the marriage of gradient-layer direct calorimetry with O_2_/CO_2_ respirometry, to simultaneously measure total heat dissipation and to estimate aerobic heat production (Burnett and Grobe, 2013, 2014). Rodents undergo surgical implantation of a core temperature telemeter, recover for at least 1 week, and are then studied in the calorimetry chamber while at rest.

In yet another example, to further explore the effects of ventilatory and chemosensory functions on cardiometabolic phenotypes, animals may be studied *via* unrestrained whole-body plethysmography. Animals are placed into a chamber that resembles a respirometry chamber and animals are then serially exposed to ambient air mixtures with altered and known O_2_ and CO_2_ content to evaluate breathing parameters and metabolic rate responses to hypoxic and hypercapnic conditions.

Finally, our team offers methods to assess blood pressure and cardiovascular autonomic functions in rodents though instrumentation with radiotelemetric blood pressure transducers. Blood pressure waveforms recorded at very high frequency can be analyzed to assess systolic, diastolic, and derived endpoints such as mean pressure, along with heart rate, and then secondary analyses can be performed to estimate autonomic functions, *via* spectral power and heart rate variability analyses. Subsequently, more robust studies of autonomic function can also be performed by assessing pressure and heart rate responses to the acute injection of pressor agents.

Regardless of the combination of experimental endpoints utilized by an investigator, the CRMPC and collaborating team provide expert assistance in data archiving, analysis, presentation, and statistics. Below follow more detailed descriptions of each major technology and technique.

## Individual Methodologies

### Time-Domain Nuclear Magnetic Resonance Spectroscopy

Analysis of body composition is the first step in comprehensively assessing metabolic and fluid homeostasis phenotypes in rodents and is especially useful when performing studies investigating pharmacological, genetic, or dietary manipulation of the animal model where a body mass phenotype manifests. Understanding what constitutes the body composition of the animal model provides a number of useful insights into a variety of bioenergetic components that ultimately define overall body weight.

There are several methods available for quantification of tissue and fluid masses, such as dual energy *X*-ray absorptiometry (DXA), magnetic resonance imaging (MRI), and time-domain nuclear magnetic resonance spectroscopy (TD-NMR), each possessing advantages and disadvantages. For instance, a major advantage the DXA, MRI, and TD-NMR technologies all share is the ability to determine body composition serially/longitudinally in an animal over time with relatively minimal detriment to the overall health of the animal. However, with this in mind, a major drawback that clearly separates these technologies is that DXA and MRI methodologies take longer to perform and require use of anesthesia and standardized animal positioning in order to obtain usable scans with high precision. In contrast, TD-NMR technology can avoid these drawbacks by utilizing species-specific restraints that aid in uniform positioning of the animals (near immobilization) for the measurement scanning while also not requiring anesthesia which is clearly beneficial to the overall health of the animal. TD-NMR and MRI machines generate strong magnetic fields to acquire and analyze proton signals from the animal to determine body composition; thus, no metal can be introduced into the magnetic fields during the measurement scanning. This is a major drawback that precludes use of animals with metal implants, such as radiotelemetric implants, minipump implants, and electrodes, which must be a consideration in the experimental design. Lastly, DXA and MRI technologies possess the ability to generate images of the fat mass distribution in the rodents while TD-NMR does not, and DXA can enable quantification of bone structure, which neither MRI nor TD-NMR can achieve, and thus, a combination of these methods may be beneficial for the investigator depending on the questions of interest.

**Figure 3:**
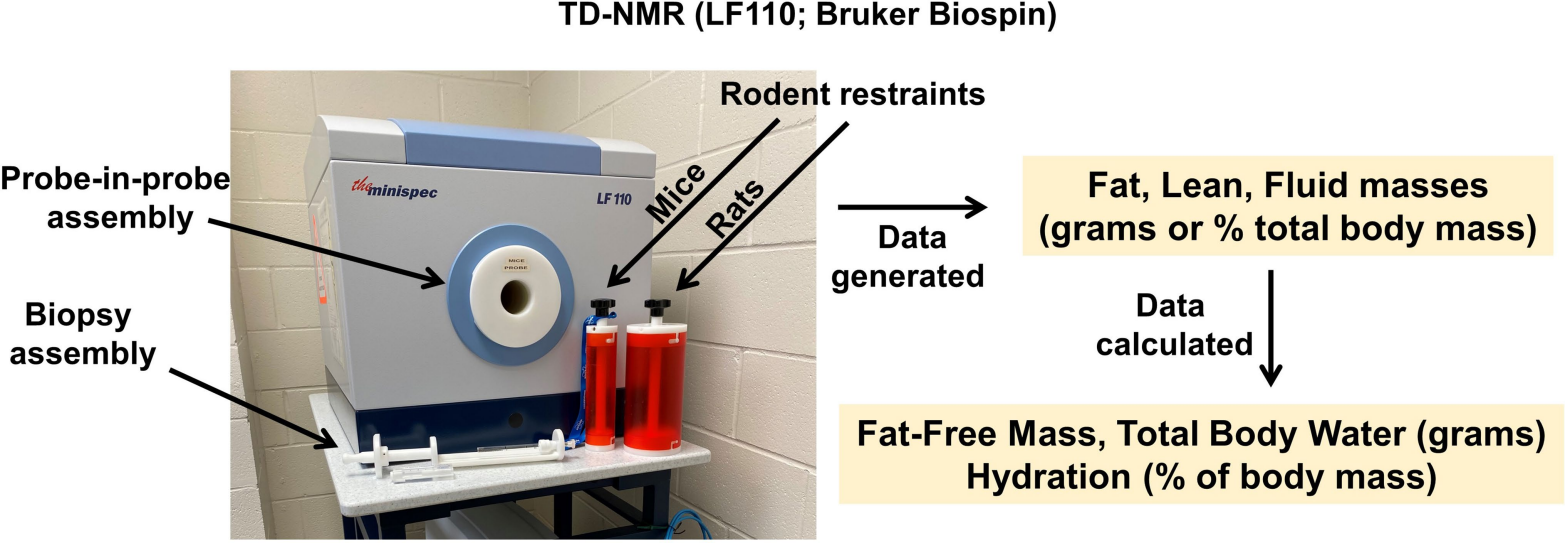
Time-domain Nuclear Magnetic Resonance (TD-NMR) body composition analyzer. Bruker model LF110 TD-NMR with multiple antenna probe assemblies, which permits analysis of tissue biopsies, mice, or rats with great precision from the same instrument, which reduces space requirements without major reductions in resolution across a wide range of sample sizes (e.g., 0.05–1,000 g).

The CRMPC utilizes TD-NMR technology for body composition analysis of rodents as it provides rapid (<2 min/measurement), non-invasive (light restraint), and longitudinal (non-terminal) /serial analyses in both mouse and rat models. Specifically, the CRMPC utilizes the newest generation of LF TD-NMR analyzers, the LF110 (Bruker Biospin; Billerica, MA; Figure 3). One of the many advantages of the LF110 is the overall mass range of biological samples that can be measured in the system that utilizes a probe-in-probe design to keep the NMR footprint at a minimum (41″ × 28″ × 57″) as opposed to housing two separate systems to measure mice and rats, respectively. The LF110 possesses the ability to quantify with precision small tissue samples (such as mouse fetuses or organ biopsies; 10–500 mg), various sizes of mice (up to 130 g), and large rats (up to 1 kg). The LF110 has sufficient resolution for this wide range of body compositions because of its ability to generate sufficiently high magnetic fields that are both safe to the animal and operator and accurate within 1% of total body mass. Upon completion of a scan, the Bruker LF110 TD-NMR directly provides empirical measurements of fat, lean, and fluid mass (expressed in grams) as well as the calculated percentages of these body composition components of the total body mass of animal based on the body weight entered by the operator.

As stated above, the data generated by the LF110 NMR can provide useful information for users on body composition and energy requirements for the observed body mass phenotype as well as fluid balance in a variety of biological conditions such as genetically manipulated rodents, studies of diet-induced obesity, and others. Subsequent endpoints can also be calculated from TD-NMR data as follows. Whereas the “fat” mass reported by TD-NMR corresponds to hydrogen ions associated with large molecules (i.e., fatty acids), this value strongly corresponds to the fat mass of the animal; in contrast, the “lean” and “fluid” masses reported by TD-NMR are reflective of hydrogen ions that are associated with medium−/small-sized molecules, and essentially-free ions, and therefore, their biological equivalent is difficult to interpret. Free-fat mass (FFM), a more biologically relevant endpoint, which is not the same variable as “lean” mass as reported by the TD-NMR, is simply calculated by the subtracting total body mass (measured in g) from the fat mass (measured in g):


$$\left[\mathrm{Fat}\;\mathrm{Free}\;\mathrm{Mass}\right]=\left[\mathrm{Total}\;\mathrm{Body}\;\mathrm{Mass}\right]-\left[\mathrm{Fat}\;\mathrm{Mass}\right]$$

This FFM value is commonly utilized to normalize food intake and energy expenditure endpoints (Pontzer et al., 2021). Further, FFM data are useful to assess fluid and electrolyte endpoints. In healthy post-adolescent mammals, many studies have established that FFM is comprised of 73.2% water (Sheng and Huggins, 1979; Segar et al., 2021). Thus, total body water (TBW; expressed in grams or milliliters if specific gravity is assumed to be 1.000) is simply calculated as 73.2% of the FFM for rats and mice. (Notably, other constants are appropriately applied for other animals and should be confirmed by desiccation approaches where possible):


$$\left[\mathrm{Total}\;\mathrm{Body}\;\mathrm{Water}\right]=73.2\%\left[\mathrm{Fat}\;\mathrm{Free}\;\mathrm{Mass}\right]$$

Finally, “hydration” is a concept that reflects the amount of the total mass of an animal that is water. Thus, the hydration of an animal is simply the fraction (i.e., percent) of the total body mass that is water:


$$\left[\mathrm{Hydration}\right]=\frac{\left[\mathrm{Total}\;\mathrm{Body}\;\mathrm{Water}\right]}{\left[\mathrm{Total}\;\mathrm{Body}\;\mathrm{Mass}\right]}$$

A typical lean rodent is ≈60% hydrated, while conditions such as obesity progressively reduce total body hydration, due in part to a proportional expansion of the fat mass (Segar et al., 2021).

#### Advantages and Disadvantages

Major advantages of TD-NMR include its precision and therefore utility for repeated measurements, the speed of analysis and therefore, animals must only be briefly restrained but not anesthetized. Relative to other technologies such as DXA, the major disadvantages are the inability to assess bone mass or density, and the inability to analyze animals that are instrumented with metallic implants, such as radiotelemeters or minipumps.

### Bioimpedance Spectroscopy

Another technology that has been advanced to provide rapid assessments of body composition involves bioimpedance. This approach involves passing a current through the body at one or more frequencies (i.e., bioimpedance analysis (BIA), multifrequency BIA (MF-BIA), or bioimpedance spectroscopy (BIS)). The major advantage of this approach is that in addition to assessing body fat, it also provides an assessment of body fluid compartmentalization (i.e., extracellular versus intracellular reservoirs). Unfortunately, when applied to rodents, the accuracy and precision of this approach is modest; however, our team has recently demonstrated that when combined with TD-NMR, the accuracy of this method is vastly improved such that exceptionally small volumes of fluid compartments can be assessed in rodents (Segar et al., 2021). This non-invasive combinatorial method therefore represents a breakthrough, as classic methods to determine body fluid reservoirs include injection of tracers and/or terminal studies, which represent possibly unacceptably invasive methods.

**Figure 4:**
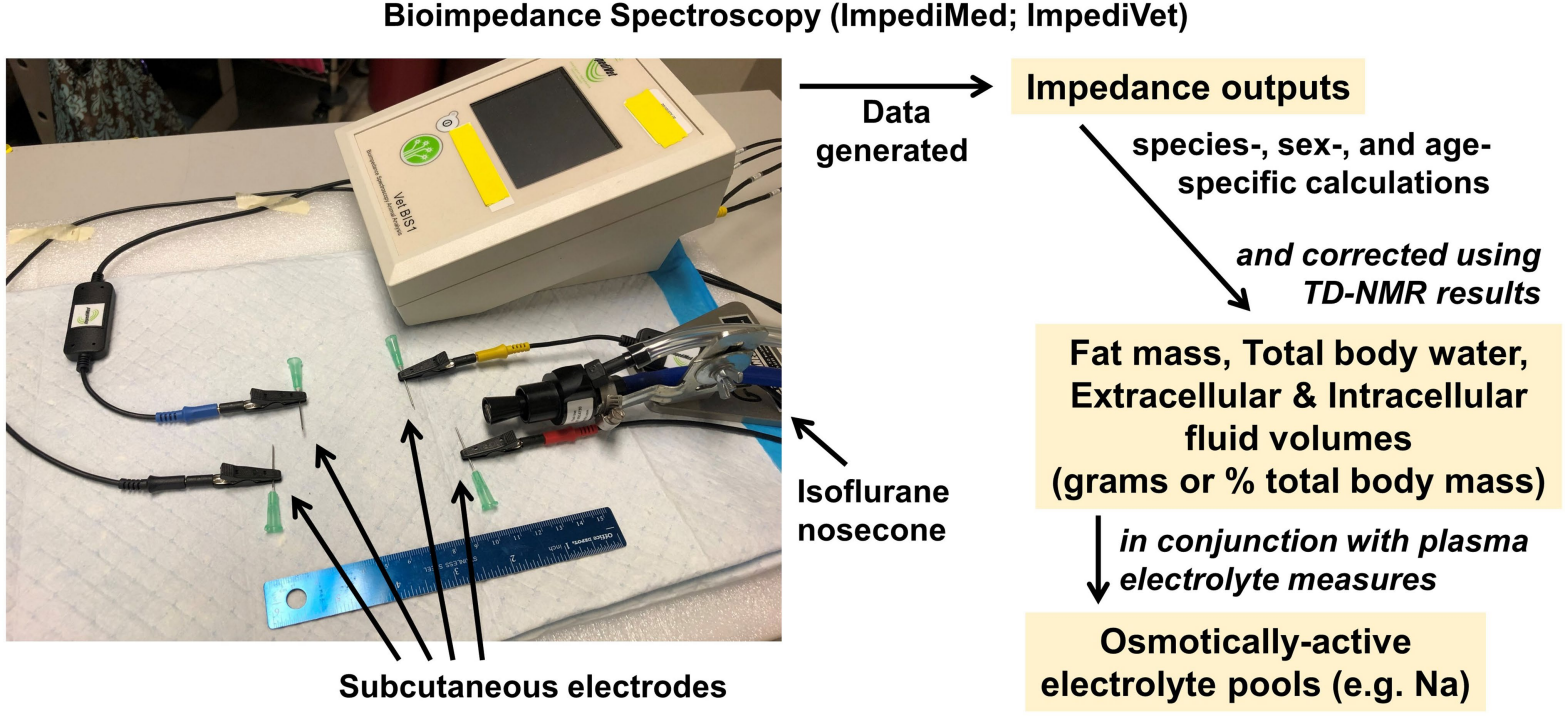
Bioimpedance spectroscopy analyzer. ImpediMed ImpediVET BIS1 Bioimpedance Spectrometer, which when used in combination with TD-NMR, provides rapid, minimally invasive assessments of fluid compartmentalization. Assessments of fluid compartment volumes can then be used in conjunction with assessments of electrolyte concentrations in these compartments to calculate whole-body osmotically active electrolyte pools.

To perform NMR/BIS, body fat stores are first quantified by TD-NMR, and then, mice are anesthetized by inhalant, such as isoflurane. Subcutaneous electrodes are implanted in four specific positions on the bridge of the nose, the top of the head, the lower rump, and tail of the animal. A carefully calibrated electric current is briefly passed through the animal using an ImpediVet system (ImpediMed; Carlsbad, CA; Figure 4), and the resistance to the current is interpreted by the system to assess fat mass, extracellular, and intracellular fluid masses. Three individual assessments are performed per animal, which requires a total of approximately 5 min. Animals are then allowed to recover from the anesthesia and returned to home cages, or blood collections are performed as detailed below under the section on electrolyte analyses.

Importantly, as recently described in detail (Segar et al., 2021), raw outputs from BIS analyses from rodents are then corrected using fat mass data determined from TD-NMR analyses performed immediately before the BIS analyses. Briefly, from the BIS outputs, the relationship between the “distance between electrodes” variable input into the BIS system during analysis and resulting “BIS-derived fat mass” values is determined by linear regression, and the “true distance” is calculated by interpolation from the “TD-NMR-derived fat mass.” This “true distance” value is then used to interpolate the corrected extracellular and intracellular fluid values from the BIS dataset. Taken together, this novel combinatorial methodology can provide investigators rapid, minimally invasive, and high-resolution assessments of fluid compartmentalization in small rodents.

#### Advantages and Disadvantages

Relative to other methods of assessing fluid compartmentalization, the major advantages of the BIS approach are its speed, which enables rapidly repeated measures and thereby kinetic assessments, and its minimally invasive nature, as nothing must be injected into the animal that might have its own physiological consequence (such as the introduction of fluid volume or compounds that might have osmotic or other effects on its own). The major disadvantage of this method is the fact that it must be performed during anesthesia, even though that anesthesia only lasts a few minutes.

## Metabolic Caging

**Figure 5:**
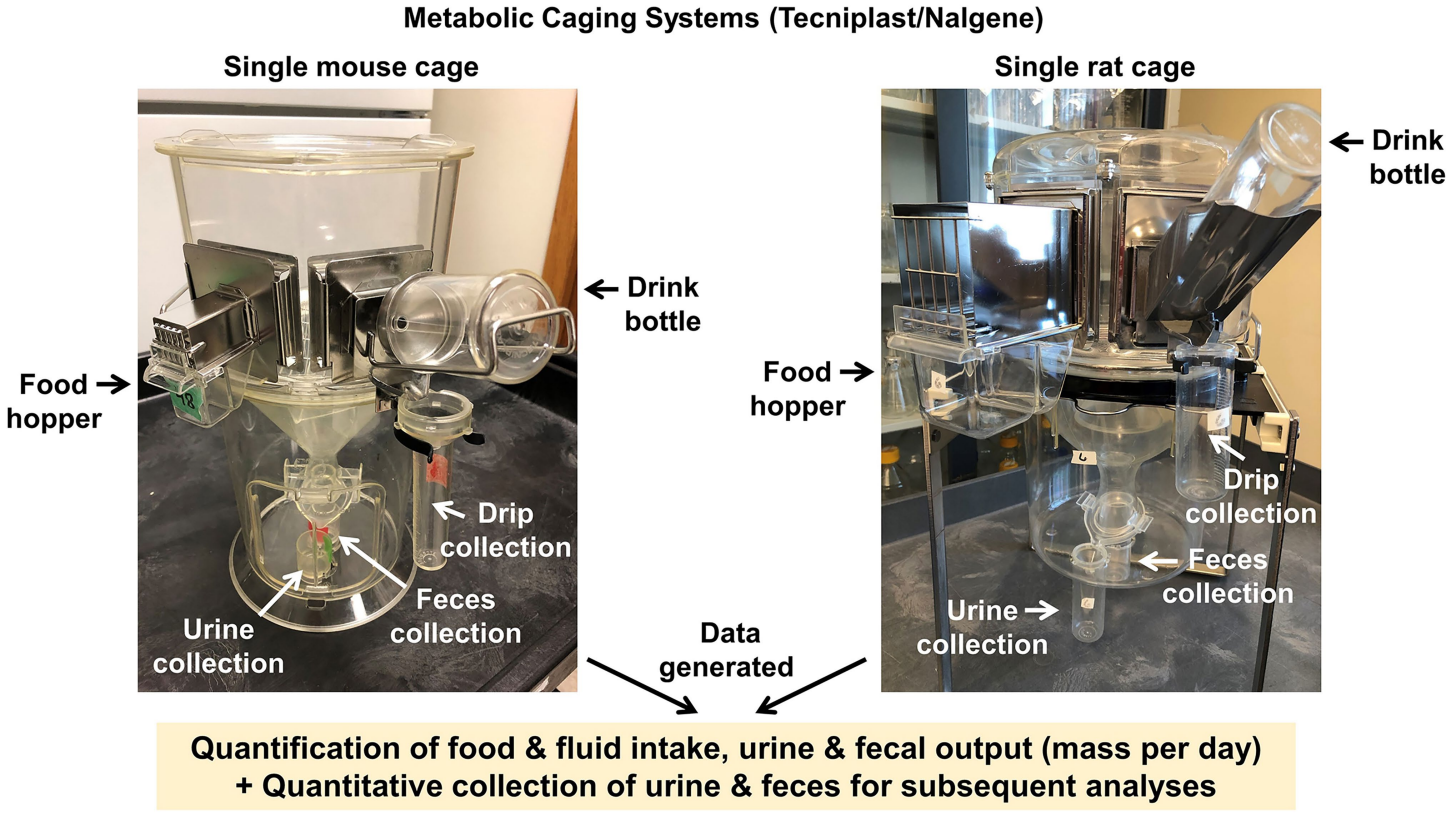
Metabolic caging systems. Tecniplast/Nalgene style metabolic caging systems for individually housed mice and rats, which permit quantitative assessments of food and fluid intake, and quantitative assessments and collection of urine and fecal outputs.

Metabolic caging offers the investigator a means for quantitative assessments of food and fluid intake in conjunction with urine and fecal output measurements and collection in a single housing setup. The CRMPC provides Tecniplast/Nalgene single-animal metabolic caging (Mouse model #3600 M021; Rat model #650-0100 and #650-0350) (Grobe, 2017) (Figure 5).

We employ a general paradigm for metabolic caging studies where animals are subjected to TD-NMR body composition assessments prior to the start of metabolic cage studies. Animals are then placed into metabolic cages (i.e., on a Monday) and food intake, water intake, fecal production, and urine outputs are measured every 24 h for several days (i.e., until Friday). We recommend to our investigators that collections for downstream applications such as bomb calorimetry and flame photometry are collected on Thursday and Friday (i.e., overnight periods “3” and “4”) of the metabolic cage paradigm, as at least the first 2 days represent a critical acclimation period for the animals to this type of housing (Grobe, 2017). It is important for an investigator to understand that rodents housed in metabolic caging with wire flooring demonstrate considerable variability in body mass stability, food/water intake, and urine/feces production due to the initial stress of the housing apparatus and inability of the rodents to utilize thermoregulatory behaviors, such as huddling, nesting, and burrowing, as they might otherwise do in home cages. However, proper acclimation to the metabolic cages has been shown to bring more consistency to these physiological variables. Indeed, previous studies from our group have demonstrated ~3–5 days of acclimation to metabolic cages generally cause mice to exhibit a plateau in body mass and intake behaviors (Grobe et al., 2010; Grobe, 2017; Ziegler et al., 2022). However, it is important to reiterate that individual investigators need to experimentally determine the best acclimation protocol for their own animal model of interest, and note that longer acclimation time periods may be needed depending on strain/age/sex/genotype, etc. Further, investigators may consider placing metabolic cages inside of environmental chambers with sufficient air exchanges (e.g., rodent incubators from Powers Scientific, Inc., Pipersville, PA) maintained at warmed ambient temperatures to offset the presumed thermal challenge imposed by maintaining animals on wire grid floors. Investigators would need to carefully consider and justify the choice of any specific ambient temperature exposure; however, as the lower and upper critical temperatures (i.e., thermoneutral zones) differ between species and need to be established within individual strains and genetically modified strains. Upon removal of the animals from the metabolic cages at the end of the study period, TD-NMR measurements are repeated to determine body composition changes over the course of the study.

**Figure 6:**
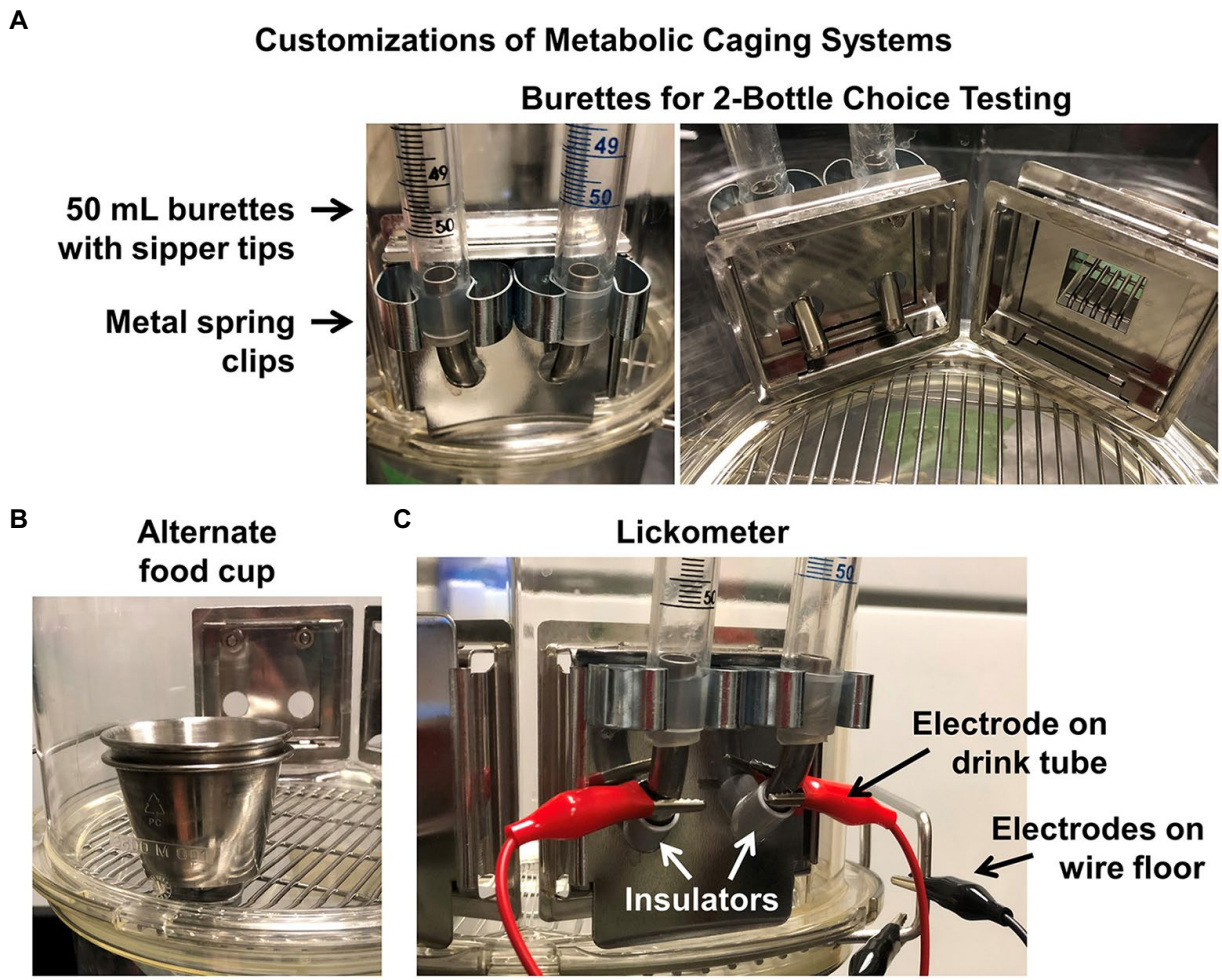
Customizations to metabolic caging systems. Example customizations to metabolic cages, such as **(A)** instrumenting cages with two 50 ml burettes to permit 2-bottle choice testing, **(B)** adding a food cup/hopper within the cage to hold pellets, pastes, or slurries that are incompatible with the standard hopper design, and/or **(C)** instrumenting drinking tubes with differential electrodes to serve as a voltage-based lickometer.

The metabolic caging protocol described above is useful for standard collections using powdered chow diets and a single liquid source but does not allow for more complex study paradigms such as multiple drink choices (e.g., water versus salt or sucrose solutions). Our team has modified the standard, commercially available metabolic cage design to accommodate presentation of a variety of fluids and foods (Figure 6A). For example, our cages have been modified to allow 2-bottle choice paradigms using 50 ml burets equipped with standard metal sipper tube tips, to permit assessment of intake of two different solutions, and to allow movement of the burets to assess and account for side biases. Additionally, our cages can be modified with the addition of stainless-steel cup inserts that are attached to the wire cage bottom of the metabolic housing unit (Figure 6B). These cup inserts can enable investigations of intake of waxy or sticky diets such as extremely high-fat diets that can be overly cumbersome to use and time consuming to powderize and deliver *via* the standard food hopper system.

It is important to recognize that the use of metabolic caging remains a topic of ongoing concern and is subject to regulations that differ by institution and country. For example, studies of male BALB/c mice housed in metabolic cages support the conclusion that these animals did not effectively acclimate to the caging even after 21 days (Kalliokoski et al., 2013). In addition, the European Union Directive 2010/63/EU classifies the use of such caging for more than 24 h as a moderate to severe procedure (European Union Directive, 2010). Thus, there remains an ongoing need for refinements and improved approaches to quantitatively collect urine and feces from rodent models. Investigators are encouraged to consider the pros, cons, and feasibility of alternative approaches whenever possible and appropriate.

### Advantages and Disadvantages

Metabolic caging is the only widely used method currently available to quantitatively collect urine and feces over longer time periods (e.g., 24 h and beyond), and therefore, it is difficult to articulate specific advantages and disadvantages, as no effective comparator is available. Nonetheless, investigators are encouraged to understand the challenges and consequences of using this approach, including potential unintended behavioral and physiological effects on the rodent under study.

### Lickometers

To further dissect the forces governing fluid intake behaviors, one approach is lickometry. Lickometers are used to assess licking behavior from a drink bottle. Because of the rapidity of the licking event and the exceptionally small volume of exchange of individual licks from a rodent, licking behavior can be interpreted differently from gross fluid consumption data. For example, whereas a mouse may typically consume 2–4 ml of a solution per day, this consumption is influenced by both appetitive drives versus post-ingestive consequences. Licking behaviors upon approach to the bottle are dominantly governed by the appetitive aspects of the solution. Thus, employing lickometer equipment in addition to traditional metabolic caging equipment allows for further dissection of the behaviors and physiology influencing ingestive behaviors in the rodents under study. Genetic and physiological influences upon the nuanced patterns of licking behaviors in rats and mice have been identified using such approaches (St John, 2017; St John et al., 2017).

There are a few lickometer systems available commercially that utilize optical beam breaks at the water sipper to determine a drinking event. The CRMPC utilizes a custom-fabricated version of lickometers, based on previous designs from Hayar et al. (2006), which involve attaching an electrode clamp to the sipper tube tip of the water bottle or buret in a metabolic cage, as described above (Figure 6C). The other electrode is then attached to the wire cage bottom of the metabolic cage. When the rodent begins a drinking event the tongue completes the circuit which is then measured as a deflection in the voltage signal on a PowerLab, using associated LabChart Pro software (AdInstruments, Colorado Springs, CO). Data are processed within the LabChart Pro software using the Peak Analysis module, to analyze lick burst patterns.

Many additional behavioral assays are available and appropriate to further investigate the various factors influencing food and fluid ingestive behaviors, including motivation, perception, and cognition. While our collaborating laboratories and the MCW Neuroscience Research Center (NRC) Rodent Behavior Core facility are well equipped to investigate these endpoints, review of these aspects and approaches is beyond the scope of the current review.

#### Advantages and Disadvantages

Relative to other lickometer designs, such as those that use photoelectric detectors, the primary advantage of our system stems from the fact that this system is a simple addition to the existing metabolic caging system in use by our team. As a result, lickometer measures can be simultaneously and non-invasively performed in animals that are concurrently studied for food and fluid intake, urine and feces production, and for urine and feces collection. The disadvantage of this setup also stems from this design, as animals are housed in the metabolic cage, which may have unintended effects on behavior and physiology, as discussed above.

### Bomb Calorimetry

Food intake/ingestive behavior is generally easy to understand and to measure; however, food ingested is not the same as the calories that are absorbed by the body and its associated microbiota. To determine the actual absorption of calories, one must also consider the efficiency of the digestive tract (i.e., digestive efficiency). Currently, there are several techniques available to determine digestive efficiency in rodents that are relatively inexpensive and simple to perform. These methods include fecal acid steatocrit (a biochemical method) and metabolomics which measure the fecal fat absorption rates to assess digestive efficiency and to understand the nuanced changes in types of fat absorption. However, these techniques are qualitative in nature and do not quantitatively determine the caloric content of the feces.

**Figure 7:**
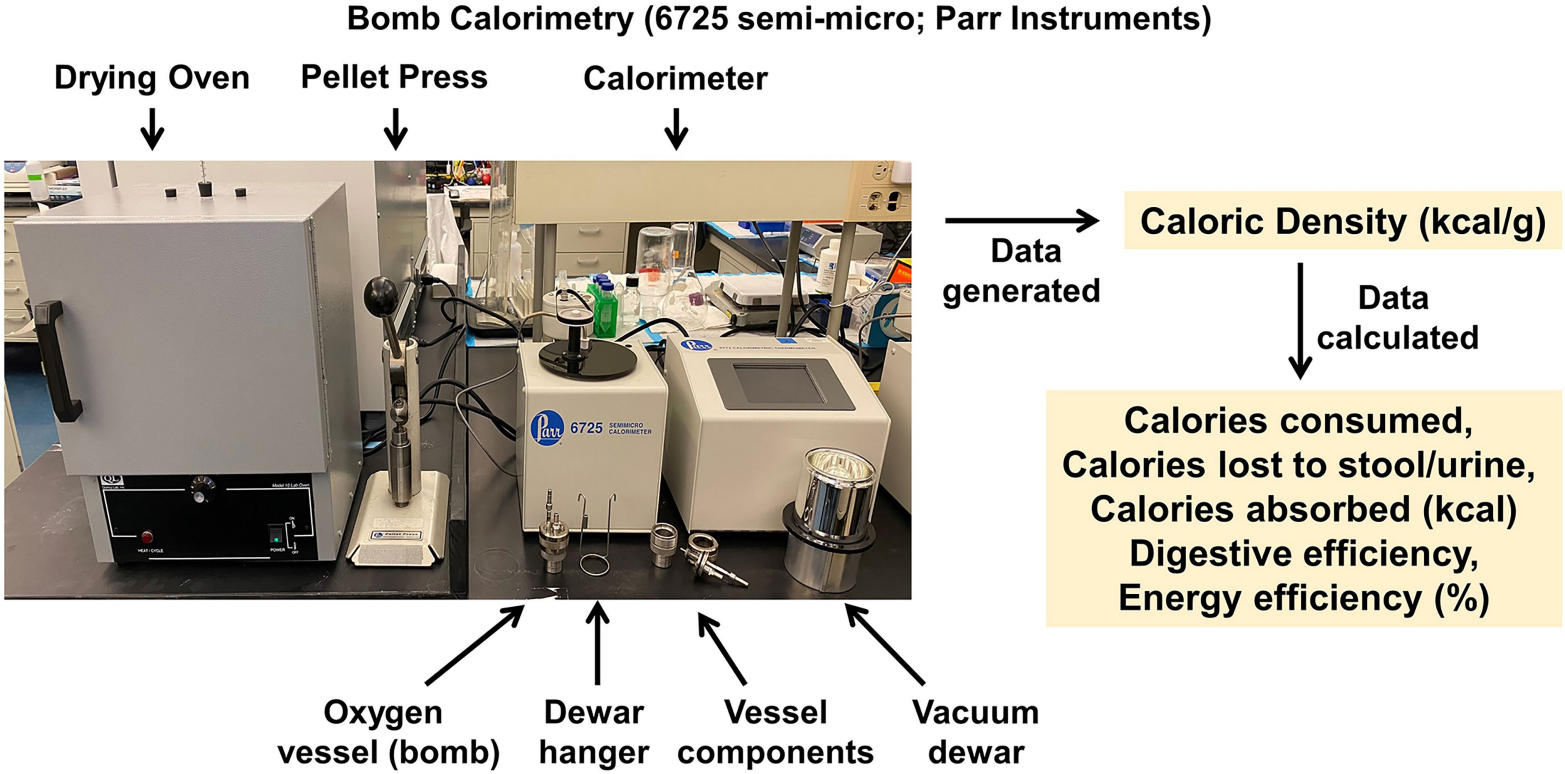
Semi-micro bomb calorimeter. Parr semi-micro bomb calorimeter system, for analysis of the energy density of food, fecal, urine, and other biological samples. When applied to samples collected in a quantitative manner (e.g., *via* metabolic caging systems), digestive and energy efficiencies can be calculated with high precision.

To enable quantitation of digestive efficiency, the CRMPC utilizes bomb calorimetry. In general, bomb calorimetry involves the measurement of the heat of combustion of a known sample (i.e., heat liberated from a sample that is combusted to completion in a pure oxygen environment). The amount of heat produced (heat energy) can then be mathematically utilized to determine the caloric density of the original sample. Because of the small sample sizes involved when assessing caloric density of urine and fecal samples from mice and small rats, we utilize a 50–200 mg semi-micro bomb calorimeter (Model 6725; Parr Instruments, Moline, IL; Figure 7). In brief, feces are collected *via* species-specific metabolic caging systems to allow for quantitative measurements of food ingested and feces produced per unit of time (i.e., per day).

Freshly collected feces are weighed to determine “wet mass” and are then desiccated in a drying oven (60°C for 3–4 days) and reweighed to determine “dry mass” of the feces. This ratio may be of interest to the investigator to determine if different diet compositions or experimental manipulations of the animals contribute to differing water content of the feces. It is important to note investigators should use an analytical balance (i.e., resolution of 0.0001 g) to provide sufficient resolution for subsequent analyses. Feces are then pressed into pellets using a pellet press (Parr Instruments), ignited using a nickel chromium fuse wire of known caloric value, and burned in a pure oxygen environment within an oxygen vessel (Model 1109A; Parr Instruments) to complete combustion. To enhance rigor and reproducibility, it is important to thoroughly determine the heat capacity or energy equivalent (EE) of each bomb and dewar combination as subtilties in manufacturing of the metal comprising the oxygen vessels exist and consistent use over time can alter this heat capacity and result in errors in accuracy. Determination of the energy equivalent can be easily achieved by thorough and frequent calibration of the oxygen vessels by burning predetermined standards (such as benzoic acid).

In addition to the caloric content of the feces, it is important to empirically measure the caloric densities of the diet fed to the animals. Most diet manufacturers commonly report the caloric density of the diet through calculating calories based on estimations of metabolizable energy from the food source using the Atwater system (estimations: 4 kcal/g to protein, 4 kcal/g to carbohydrates, and 9 kcal/g to fats). These estimations suffer considerably from lot-to-lot variations and subtle changes in the manufacturing of the diet, resulting in the reported caloric density from the vendor being substantially different from the processed caloric density in bomb calorimeters. Additionally, it is important to thoroughly remove excess water in diets to accurately determine its caloric density, especially for high-fat and other “Western” high-calorie diets that have significant water content.

In addition to feces and food samples, energy density can be determined in other biological samples from the animal, such as the urine. Energy lost to urine in rodents is a very small amount compared to the feces but may still hold significance depending on species and disease states such as in models of diabetes. For example, C57BL/6 J mice generate between 0.5 and 1 ml urine per day and utilizing bomb calorimetry we have determined mice lose roughly 0.01 kcal/ml to urine. Thus, these animals lose ≈0.005 kcal/d to urine, compared to a total daily flux of ≈10 kcal/d through the body (0.05%). However, in rats which generate ≈10 ml/day (though this value greatly varies by strain), we have determined that urine contains closer to ≈0.2 kcal/ml or ≈1 kcal/day (which again, varies by strain). Compared to a total daily flux of 50–75 kcal/d in adult rats, this indicates that healthy rats excrete ≈1.5–2% of consumed energy to the urine. In states of glucosuria as occurs during diabetes, for example, this energy loss to urine would be expected to increase considerably. If human obesity is due to an energy imbalance of only 0.35% (Hall et al., 2011), the potential physiological significance of an energy “output” to urine of 1.5–2% or more becomes obvious.

Various calculations are made following the assessment of caloric densities by bomb calorimetry, as follows. First, calories consumed is determined as:


$$\begin{array}{l} \left[CaloriesConsumed\right]=\left[FoodMassConsumed\right]\times \\ \;\;\;\;\;\;\;\;\;\;\;\;\;\;\;\;\;\;\;\;\;\;\;\;\;\;\;\;\;\;\;\;\;\;\;\;\;\;\;\;\left[CaloricDensityofFood\right] \end{array}$$

Water lost to feces can be calculated after feces are desiccated for 4 days at 60°C, which may be of interest to calculations of fluid balance in some animal models:


$$\begin{array}{l} \left[WaterLosttoFeces\right]=\left[Wet\;MassofFeces\right]-\\ \;\;\;\;\;\;\;\;\;\;\;\;\;\;\;\;\;\;\;\;\;\;\;\;\;\;\;\;\;\;\;\;\;\;\;\;\;\;\;\left[Dry\;MassofFeces\right] \end{array}$$

Then, the calories lost to stool are calculated as:


$$\begin{array}{l} \left[CaloriesLosttoStool\;\right]=\left[MassofFecesProduced\right]\times \\ \;\;\;\;\;\;\;\;\;\;\;\;\;\;\;\;\;\;\;\;\;\;\;\;\;\;\;\;\;\;\;\;\;\;\;\;\;\;\;\;\;\;\left[CaloricDensityofFeces\right] \end{array}$$

From these values, several important additional endpoints can be computed. First, one can calculate the total calories that were absorbed by the animal which must be “accounted for” by expenditure or growth:


$$\begin{array}{l} \left[CaloriesAbsorbed\right]=\left[CaloriesConsumed\right]-\\ \;\;\;\;\;\;\;\;\;\;\;\;\;\;\;\;\;\;\;\;\;\;\;\;\;\;\;\;\;\;\;\;\;\;\;\;\;\left[CaloriesLosttoStool\right] \end{array}$$

As discussed above, it may be important in rats and in models of diabetes to also include a term for calories lost to urine. We have not yet evaluated the caloric value of secreted or other fluids (sweat, saliva, milk, etc.) in either species, but it seems unlikely that proteins and other solutes eliminated in these fluids account for as many calories per unit time as urine. Importantly, the calories lost to stool and other outputs are not necessarily the same unaltered substances that were ingested by the animal; but instead, they may represent calories lost by the animal in the form of enzymes or bile, etc., that are actively secreted by the animal to aid digestion. Regardless, these substances contain energetic bonds and represent caloric equivalents that are lost from the body as a whole and therefore factor into calculations of whole-body energetics in an equivalent manner.

Once absorbed calories are calculated; then, digestive efficiency can be calculated. This value represents a metric of the fraction of calories ingested that are absorbed by the digestive tract. In animals fed a grain-based diet, this is typically near 80–85%, versus 90–97% in animals fed refined high-fat diets, as grain-based diets contain more insoluble fiber:


$$\left[DigestiveEfficiency\right]=\frac{\left[CaloriesAbsorbed\right]}{\left[CaloriesConsumed\right]}$$

Finally, consumed calories and absorbed calories can be used to calculate metrics of energy expenditure. First, in the absence of a bomb calorimeter, investigators can utilize food intake data and body mass changes to calculate “feeding efficiency.” This is often used when food intake is measured over an extended period, along with body mass. Although easy to calculate, its interpretation is complicated because this value represents an inverse estimate of some complex combination of digestive efficiency and all forms of energy expenditure:


$$\left[FeedingEfficiency\right]=\frac{\left[\Delta \;BodyMass\right]}{\left[\Sigma \;CaloriesorMassConsumed\right]}$$

When a bomb calorimeter is available to determine digestive efficiency and to clarify the fraction of calories consumed that are absorbed, then investigators can account for the effect of digestive efficiency within the feeding efficiency calculation, and the resulting calculation is referred to as “energy efficiency.” This calculation again uses measures of food intake, digestive efficiency, and body mass over extended periods:


$$\left[EnergyEfficiency\right]=\frac{\left[\Delta \;BodyMass\right]}{\left[\Sigma \;CaloriesAbsorbed\right]}$$

Critically, while the use of these methods is sensitive and non-invasive, the interpretations of feeding efficiency and energy efficiency are limited. First, absolute values of these ratios should not be directly compared between studies, as ages, sexes, masses, and a multitude of environmental factors can affect the absolute values of the ratios; instead, all control and experimental groups should be included in a given cohort under study. Second, deflections in feeding or energy efficiency imply changes in total energy expenditure during the timeframe under study but do not provide any information regarding the form of that energy expenditure. For example, a reduced energy efficiency suggests increased energy expenditure but could involve increases in RMR, physical activity, the specific dynamic action of food (also known as the thermic effect of food), adaptive thermogenesis, etc., or it may involve “normal” energy expenditure in the context of a generalized reduction in caloric absorption, which results in a disproportionate increase in expenditure relative to intake. Carefully planned experimental conditions (such as feeding/postabsorptive/fasted state, etc.) and other methods are therefore necessary to dissect these individual contributions. Finally, the interpretation of both feeding and energy efficiency are complicated by time. When applied to timeframes that are too short (i.e., hours), these calculations break down because food consumed has not passed through the digestive tract and thus, the ratio is not reflective of the food consumed in the observation period. Conversely, when applied to timeframes that are too long (i.e., weeks), these calculations are confounded by compensatory changes in energy balance mechanisms. In contrast, these methods are strongly informative when applied to 1- or 2-week-long studies immediately following surgical or dietary interventions in rodents (Riedl et al., 2021).

#### Advantages and Disadvantages

Compared to other qualitative methods of assessing aspects of digestive efficiency, such as fecal acid steatocrit, bomb calorimetry offers a quantitative approach to tracking caloric flux with exceptionally high resolution. Disadvantages of this approach stem from the need to collect feces over extended periods, and by extension, the inability to dissociate time-dependent (e.g., diurnal or feeding state) effects.

### Electrolyte Assessments

**Figure 8:**
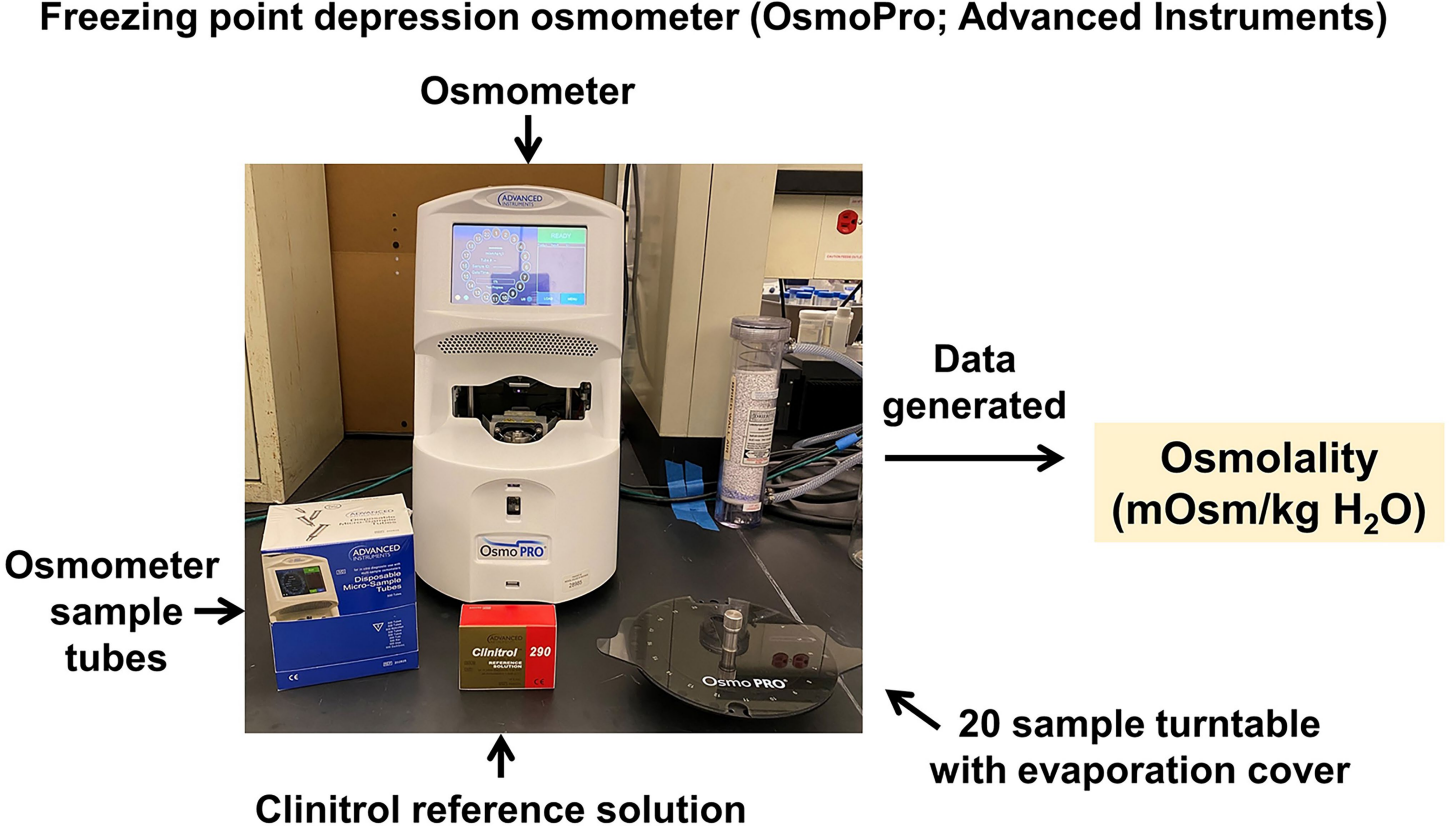
Freezing-point depression osmometer. Advanced Instruments OsmoPro freezing-point depression osmometer system, for analysis of the osmolality of fluid samples.

The CRMPC offers a variety of technologies to analyze electrolytes in samples including blood, plasma, serum, urine, feces, milk, tissue samples, or whole bodies. We utilize an array of techniques including ion-selective electrode-based methods, freezing-point depression osmometry, and flame atomic absorption spectroscopy. Measurements of total solute/ion concentration in biological samples, such as urine and blood, can provide an investigator with valuable information on normal cellular function in their sample of interest. Cellular function can be greatly altered when extracellular solute concentrations rise or fall against a threshold level. To this end, the CRMPC utilizes the gold standard methodology of freezing-point depression osmometry (OsmoPro; Advanced Instruments; Norwood, MA) for the assessment of osmolality in biological samples (Figure 8). Freezing-point depression osmometry is a superior technology compared to permeable membrane and vapor pressure osmometry as this technique offers high accuracy and reproducibility and is very simple for the user to operate. The OsmoPro utilizes a 20-sample turntable loader only requiring 20uL of sample, which is clearly beneficial for samples lacking appreciable volume, such as blood samples from mice and small rats. The OsmoPro provides rapid osmolality assessments (<2 min per sample) and a detection range of 0–2000 mOsm/kg H_2_O which is ideal for rodent biological samples. Additionally, we recommend investigators use a reference standard such as Clinitrol (Advanced Instruments) be measured along with experimental samples to assess efficacy of the calibration every run, to ensure rigor and reproducibility. Measurements of osmolality are a critical first step in uncovering alterations in urine concentrating mechanisms, renal function, and circulatory homeostasis in an investigator’s animal model of interest.

**Figure 9:**
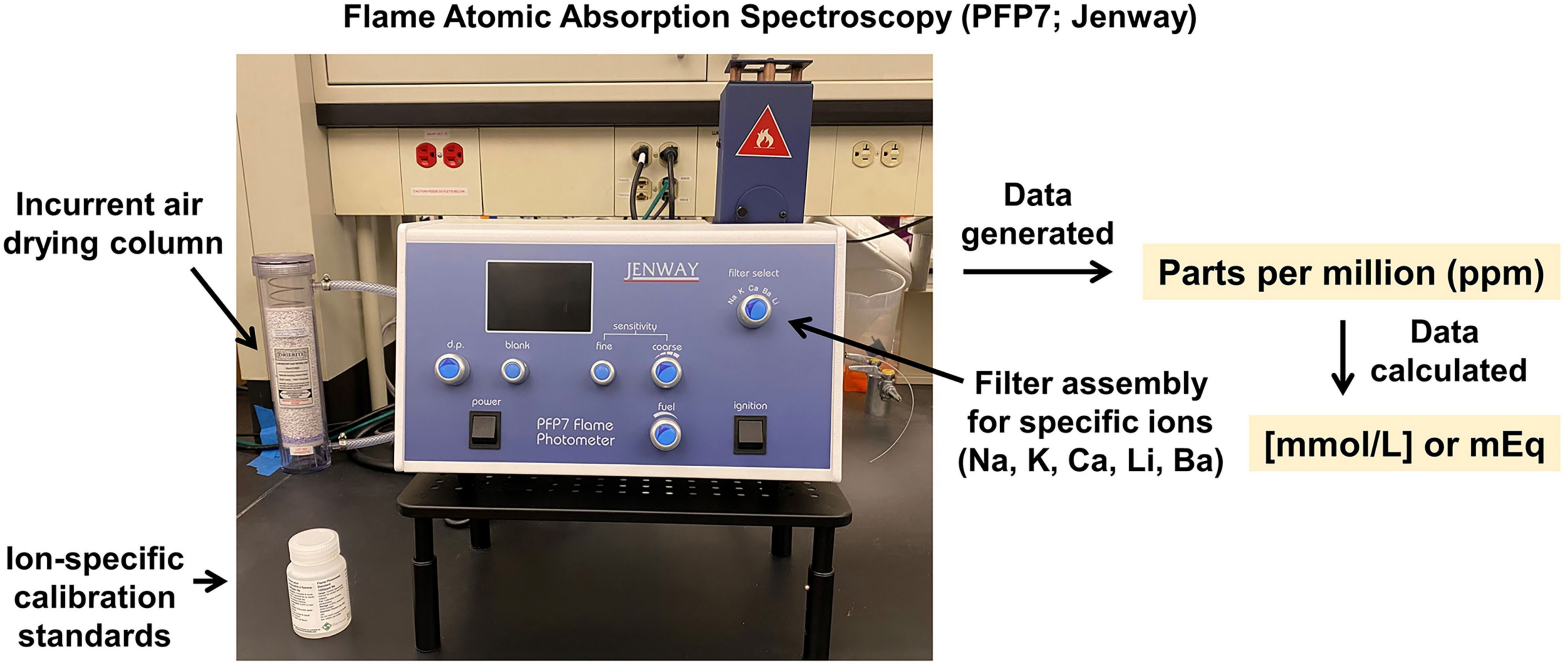
Flame atomic absorption spectrophotometer. Jenway PFP7 flame atomic absorption spectrophotometer, for analysis of sodium, potassium, lithium, calcium, and barium content of fluid samples (e.g., plasma, serum, and urine), or liquid extracts from solid samples (e.g., ashed tissues or feces).

Additionally, the CRMPC offers flame atomic absorption spectroscopy to assess concentrations of specific electrolytes (such as sodium, potassium, and calcium) in biological samples as well as tracer-based studies utilizing lithium or barium. These measurements are accomplished using a PFP7 flame photometer (Jenway) which possesses a lower detection limit for Na and K of ~5 μmol/l (Figure 9). After generation and validation of a standard curve using ion-specific calibration standards, biological samples (~100 μl volume required) are diluted 1:1000–1:7500 in deionized water and aspirated into the flame photometer. Data are then interpolated into the ion-specific standard curve and total concentration (measured in mmol/L) can be determined.

In addition to analysis of electrolytes in typical samples such as blood and urine, we have established an ashing protocol to enable electrolyte analyses across a variety of solid biological samples such as fecal samples and diets, as well as animal carcasses upon euthanasia, based on original studies by Titze and colleagues (Titze et al., 2003; Schafflhuber et al., 2007; Wiig et al., 2018). In brief, samples for ashing are placed into a drying oven (DR200, Hi-temp oven; Yamato Scientific) at 60°C for 4–5 days. Measurements of wet (day 0) and dry (after 4–5 days) weights are recorded to assess water content of the biological samples, and this is important for the calculations of hydration, and sodium and potassium content of the original sample. The temperature is then increased in a stepwise fashion to 190°C for 1 day, to 450°C for 1 day, and finally to 600°C for 2 days. The ashes are then reweighed and reconstituted in 10% nitric acid (deionized water as diluent) and then processed through the flame photometer for electrolyte analyses as described above. We and others have determined that analysis of electrolyte contents in matched urine and fecal samples from rodents indicates that fecal elimination of sodium and potassium are surprisingly large and subject to hormonal control (Nakamura et al., 2018), prompting reconsideration of conclusions drawn regarding sodium intake in studies which only measure sodium elimination to urine.

Finally, the CRMPC supports approaches to assess whole-body osmotically active and -inactive sodium reservoirs that complement and dissect the assessment of whole-body total sodium stores through ashing, as described above. After determination of extracellular (ECF) and intracellular (ICF) fluid volumes *via* NMR/BIS (as above), and plasma sodium concentrations by iSTAT or flame photometry methods, we can calculate osmotically active Na using the following equation:


$$\begin{array}{l} \left[Osmoticallyactive\;Na\right]=\left[ECF\;volume\right]\times \\ \left[Plasma\;Na\;concentration\right]+\left[ICF\;volume\right]\times \\ \left[5\;mmol/L\right] \end{array}$$

Subsequently and upon euthanasia, the animal carcasses are ashed as above to assess total body sodium stores. Osmotically inactive sodium stores are then calculated as the difference between total sodium stores and osmotically active stores:


$$\begin{array}{l} \left[Osmoticallyinactive\;Na\right]=\left[Totalbody\;Na\right]-\\ \;\;\;\;\;\;\;\;\;\;\;\;\;\;\;\;\;\;\;\;\;\;\;\;\;\;\;\;\;\;\;\;\;\;\;\;\;\;\;\;\;\;\;\;\;\;\;\;\left[Osmoticallyactive\;Na\right] \end{array}$$

#### Advantages and Disadvantages

Relative to other methods of assessing Na and K such as ion-selective electrodes and titration approaches, the use of flame atomic absorption spectrophotometry is advantageous due to its speed, repeatability, detection limits, and small sample requirement. These systems can be more expensive than other technologies, which represents a disadvantage.

Relative to other methods of measuring or estimating osmolality such as membrane or vapor pressure methods, freezing-point depression osmometry offers a simple approach that uses very small sample volumes, and its measurement is not confounded by other factors such as volatile compounds. Although osmolality can be estimated based on measurements of individual osmolytes, the difference between such estimates and measured values (e.g., the osmolality gap) can be large and variable depending on manipulations to the subject.

### Sterile Feces Collection and Fecal Material Transplant

There is increasing evidence for the significant role of the gut microbiome in the regulation of energy balance, and dysregulation of the gut microbiota has been implicated in a variety of diseases, such as obesity and cardiovascular-related disorders like hypertension (Bahr et al., 2015; Riedl et al., 2017, 2021).

The CRMPC provides assistance in these studies by providing sterile feces collections for the assessment of gut flora. Rodents are placed directly into autoclaved beakers for the collection of sterile feces by capturing them by the base of the tail (1 L beaker for mice; 5 L beaker for rats). Rodents are placed into sterilized beakers for a maximum of 2 h to collect non-contaminated feces (i.e., free of bedding and other environmental contaminants present in home caging). Fecal samples are collected by sterilized (autoclaved) tweezers (Model 12-000-157; Fisher Scientific) and placed into sterilized collection tubes. If rodents do not produce feces during the allotted time period, a sterile cotton swab wetted with autoclaved warm deionized water is used to massage the anus to promote voiding of the bowel. The fecal samples are then immediately frozen in liquid nitrogen and stored at −80°C until further analysis.

We also provide support for studies of the gut microbiome through fecal material transplant (FMT) procedures. Gavage tubes are available commercially, including both traditional metal and newer, flexible plastic types. Metal tubes are reusable after cleaning and sterilization but are nonetheless not recommended, due to increased technical difficulty of use and the potential for esophageal injury, as these needles are not flexible. We instead recommend plastic feeding tubes (mice: 20 gauge, 30 mm long; rats: 15–18 gauge, 38–100 mm long; Instech), as these gavage tubes are relatively inexpensive, simple to use, arrive pre-sterilized, and generally result in reduced trauma to the airway and throat. The FMT material is prepped daily as previously described by our team (Bahr et al., 2015). Briefly, fecal pellets to be used as FMT material from the donor cohort are collected and immediately frozen in liquid nitrogen and stored at −80°C until future use. The donor fecal pellets are pooled into a single sample and ground into a powder in a sterile mortar and pestle, homogenized in sterile PBS, and centrifuged for 5 min at 1,000 × *g* to remove large particulates. The supernatant from the resulting fecal slurry is then used for the FMT (mice: 100 μl once per day; rats: 1 ml once per day—notably, estimating and dictating numbers of bacteria in a fecal slurry are very difficult). The FMT material is loaded into the syringe, the animal is scruffed by the neck, and the plastic gavage tube is carefully placed into the esophagus and advanced into the stomach. To prevent cross contamination, each FMT treatment group receives their own plastic feeding tube(s). Studies from our team have typically involved daily gavage procedures on the animals for weeks to months (Bahr et al., 2015). Other groups have reported similar standardized methods for FMT procedures in mice (Bokoliya et al., 2021).

As an alternative to oral gavage, we have also previously used a trained oral administration method in which the FMT material is mixed into the highly palatable Nutella brand hazelnut spread, which rodents readily consume (Abais-Battad et al., 2021). The FMT material is collected as above, ground *via* sterile mortar and pestle, mixed with 1 g of Nutella, and administered orally to each individual rat. This same method can be used for FMT administration to mice using only 0.1 g of Nutella. This approach has multiple advantages and disadvantages to consider. First, relative to the use of gavage needle approaches, this Nutella-based approach reduces unintentional esophageal injury and stress. Second, this approach avoids mechanical manipulation of the neck, which must be avoided when rodents are instrumented with surgical implants such as radiotelemetric blood pressure transducers that are surgically implanted into the carotid artery, or infusion or electrode implants into the brain. In contrast, the Nutella-based method introduces potential confounding variables to the studies that must be considered and controlled, including the caloric, macronutrient, micronutrient, and electrolyte composition of the Nutella vehicle, the mass/volume/smell/taste of the Nutella, the diurnal timing of the introduction of these variables to the rodent’s environment and gut, and the various interactions among these many variables.

#### Advantages and Disadvantages

Approaches to sterile feces collection and FMT are ongoing and consensus regarding the optimal techniques to each has not been reached across this rapidly growing area of study. Here, we outline methods that we currently utilize, and our rationale for specific aspects of this approach. We encourage investigators to stay current with such methods and to empirically evaluate the consequences of proposed refinements.

### Respirometry (“Indirect Calorimetry”)

Due to its turnkey accessibility and many commercial sources, gas respirometry is the dominant technology utilized to assess energy expenditure. This method is essentially based on original empirical equivalents observed between the rates of oxygen consumed and carbon dioxide produced by an aerobic reaction and the rate of heat produced first observed by Antoine Lavosier (who also named oxygen), and later refined by luminaries including but not limited to Nathan Zuntz, William Schumburg and Graham Lusk in the early 1900’s, and then most notably Weir (1949); McLean and Tobin (1988); Lighton (2008). Weir famously provided the equation that now bears his name, relating oxygen consumption rates (VO_2_), carbon dioxide production rates (VCO_2_), and urinary nitrogen elimination rates (UN) to metabolic rate:


$$\left[VO_{2}\right]=\left[Air\;FlowRate\right]\times \left[Fractionof\;Air\;thatis\;O_{2}\right]$$

$$\left[VCO_{2}\right]=\left[Air\;FlowRate\right]\times \left[Fractionof\;Air\;thatis\;CO_{2}\right]$$

$$\left[HeatProduction\right]=3.941\left[VO_{2}\right]+1.106\left[VCO_{2}\right]-2.17\left[UN\right]$$

In this equation, if oxygen and carbon dioxide exchange rates are inserted as liters per hour, and urine nitrogen is inserted as grams per hour; then, heat production is calculated as kilocalories per hour. It is notable that few investigators measure urine nitrogen content, and thus, most choose to use the “Modified Weir Formula,” which simply represents a truncated version of the formula:


$$\left[HeatProduction\right]=3.941\left[VO_{2}\right]+1.106\left[VCO_{2}\right]$$

Critics of the Weir formula have mathematically demonstrated that when various fuel types beyond simple fats and carbohydrates such as starch or glycogen are used as fuel substrates that this equation fails to accurately calculate heat production. For example, Mansell and MacDonald showed in 1990 that when fuels, such as acetoacetate, hydroxybutyrate, protein, and ethanol, are oxidized, the equation may be importantly inaccurate (Mansell and Macdonald, 1990). More recently, Hall and colleagues proposed a refinement to the Weir equation (Hall et al., 2016), to adjust the equation according to the subject’s dietary protein intake, in which *RER* refers to the respiratory exchange ratio (i.e., VCO_2_ divided by VO_2_), and *P* refers to the fraction of calories consumed that came from protein:


$$\left[Heatproduction\right]=\left[VO_{2}\right]\frac{3.941+1.106\left[RER\right]}{1+0.082\left[P\right]}$$

Regardless of the equation employed to estimate heat production from measures of gas exchange, the primary benefit of gas respirometry when compared to other methods of estimating energy expenditure such as bomb calorimetry is increased temporal resolution. By using respirometry, minute-by-minute estimates of metabolic rate can be achieved. Thus, by calculating heat production when animals are at rest and under thermoneutral conditions, we can specifically assess RMR as opposed to activity-dependent or total metabolic rates.

**Figure 10:**
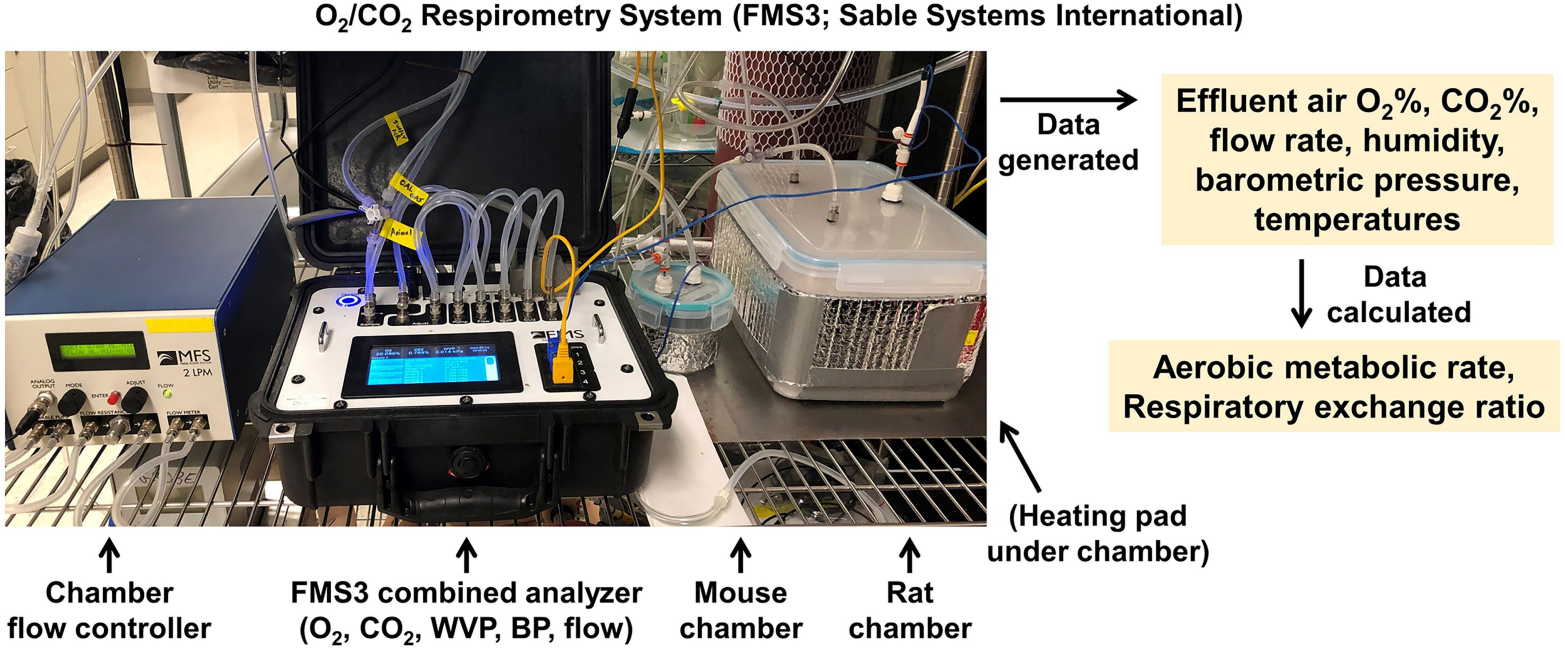
O_2_/CO_2_ respirometry (indirect calorimetry) system. Sable Systems International FMS3 gas respirometry analysis system and custom fabricated testing chambers for assessing respiratory gas exchange, and thereby estimating aerobic metabolic rate.

Within the CRMPC, we operate multiple respirometer arrays to enable assessments of RMR in rats or mice (Figure 10). Briefly, animals are individually placed into air-tight chambers appropriately sized for each species depending upon age and body size (0.05 to 1.0 L for mice and 0.5 to 8.0 L for rats), which are continuously supplied with conditioned air at a controlled rate, and effluent air from the chamber is sampled to analyze VO_2_ and VCO_2_ by the animal, along with water vapor pressure (WVP) and barometric pressure (BP; Sable Systems International, FMS3). The chambers are warmed to thermoneutral temperature ranges by electric warming pads placed beneath the chambers. The rate of air flow to the chamber is dependent upon species (300–500 ml/min for mice and 400–800 ml/min for rats; Sable Systems International, MFS-2). Animals remain in the chamber until a clear plateau in VO_2_ and VCO_2_ are observed, which corresponds with the animal resting. Testing typically lasts 4–6 h, purposefully, as this results in a 4–6 h fasting period for the animal. Critically, as thoroughly reviewed by Lighton (Lighton, 2008), the WVP dilution of respiratory gases must be considered when analyzing %O_2_ and %CO_2_ content of air, and thus, the simultaneous assessment of WVP and BP permits correction of %O_2_ and %CO_2_ as follows:


$$\left[\%O_{2_{Corrected}}\right]=\left[\%O_{2}{}_{Unadjusted}\right]\frac{\left[BP\right]}{\left[BP\right]-\left[WVP\right]}$$

$$\left[\%CO_{2_{Corrected}}\right]=\left[\%CO_{2}{}_{Unadjusted}\right]\frac{\left[BP\right]}{\left[BP\right]-\left[WVP\right]}$$

Resulting “corrected” %O_2_ and %CO_2_ composition values are subsequently utilized to calculate VO_2_ and VCO_2_ rates, which are then utilized to estimate heat production rates using Weir, modified Weir, Hall, or other equations.

#### Advantages and Disadvantages

Advantages of O_2_/CO_2_ respirometry include wide accessibility, as many manufacturers offer turnkey systems, and that these methods can easily be adapted to any cage or chamber shape or size. Additionally, the approach offers outstanding temporal resolution, which thereby enables dissociation of energy flux during periods of rest or activity. The primary disadvantages of this approach stem from the many assumptions that are required for its use, including its inability to account for nitrogen and anaerobic metabolism, and its dependence upon empirically derived formulae that have been refined several times over the last century.

### Multiplexed Metabolic Phenotyping

Multiplexed metabolic phenotyping machines are a common and effective method for rapidly assessing an array of metabolic phenotypes in rodents in a high-throughput configuration. Among the many benefits of these multiplexed metabolic phenotyping systems are the ability to simultaneously assess respirometric gas exchange, food and fluid intake, and physical activity throughout the light/dark cycle. Further, depending on the cage configuration (which varies considerably between vendors), the cage layout can be designed to resemble the dimensions and format of a typical home cage, including such design aspects as translucent plastic walls and absorbent bedding materials. Such design features are often assumed to reduce the environmental stress upon the animal and permit normal thermoregulatory behaviors such as burrowing in bedding materials. There are several vendors that manufacture multiplexed phenotyping machines including the Promethion Core (Sable Systems International; Las Vegas, NV), OxyMax/CLAMS (Columbus Instruments International; Columbus, OH), and PhenoMaster (TSE Systems; Berlin, Germany), each with argued advantages and disadvantages. Although few investigators have access to multiple competitor units, our team previously performed a comparison of a Promethion system and an OxyMax system that were located in the same room, by studying mice in a crossover design study; in general the two systems largely provided qualitatively similar results while some quantitative results significantly differed between systems (Soto et al., 2019). All such systems are quite expensive to purchase, and therefore, because investigators and institutions are likely to only purchase one system, considerable thought should be given to the design of a new system to optimize the features that are of the greatest importance to the current and future research efforts of the local team. For example, various technical aspects differ among the designs offered by each vendor, including resolution for measurements of each endpoint, nuances in cage format, flow rates, and noise.

**Figure 11:**
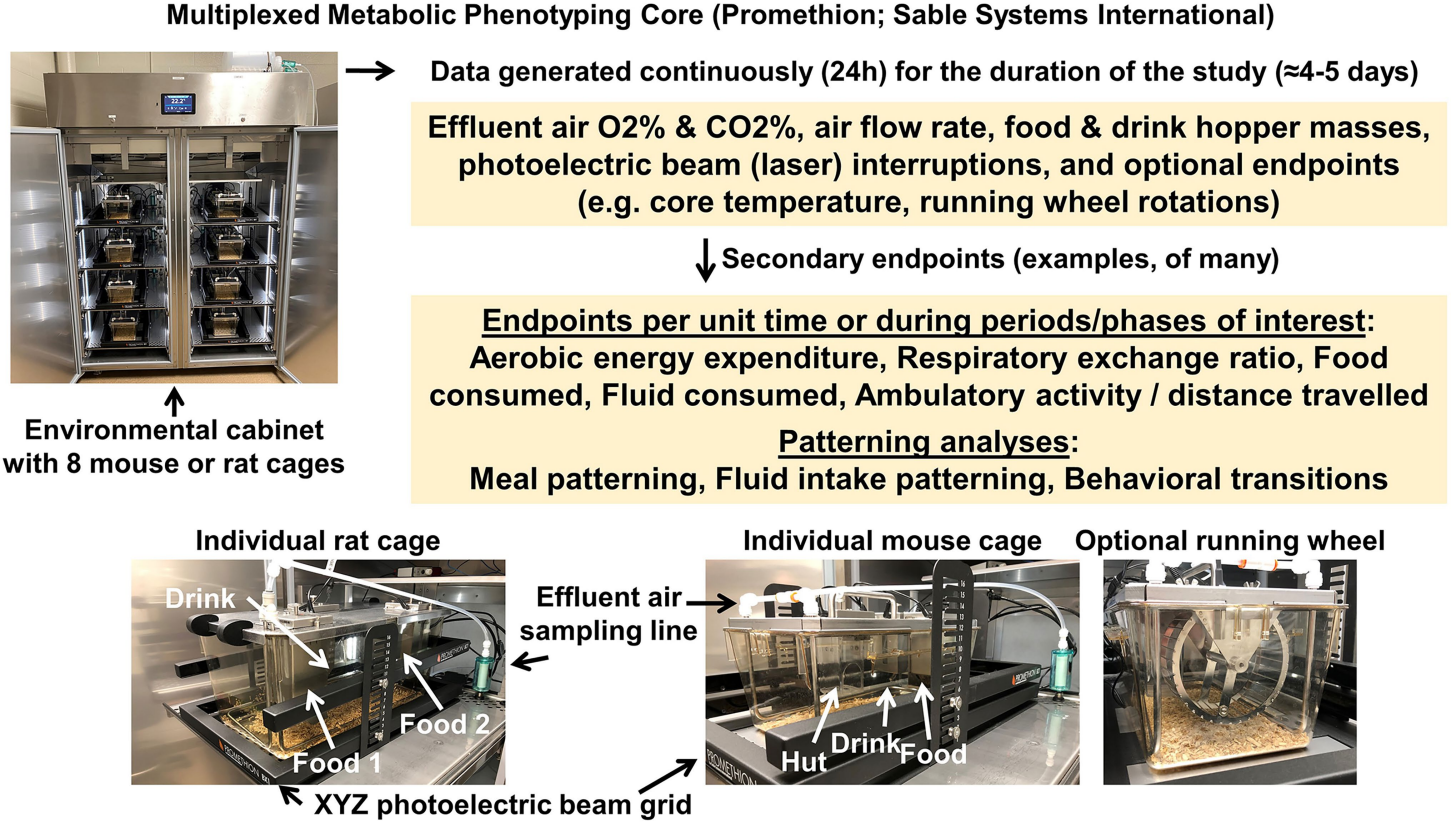
Promethion multiplexed phenotyping system. Sable Systems International 16-cage, rat-or-mouse Promethion system split between two environmental chambers (eight cages per chamber) to control ambient temperature and light.

Within the MCW CRMPC, we operate a 16-cage Promethion system spread over two environmental housing cabinets, which can be used for either mice or rats (Figure 11). Cages in this system closely resemble the ventilated cages that are utilized as home cages within the MCW animal facility, lowering the stress of the new housing environment for the animals. Because the Promethion cages are maintained inside of these environmental chambers, we can manipulate ambient temperature and lighting conditions as required for individual experiments. Our system is equipped to permit continuous measurements of the intake of food and drink, and a third optional hopper that can be equipped with either a second food or drink, or a sleeping “hut” which will permit assessment of body mass any time the animal enters. Furthermore, the system is equipped with a photoelectric (laser) grid to assess locomotor activity in X, Y, and Z axes. The system discontinuously samples air from each cage and provides a datapoint describing VO_2_ and VCO_2_ from each cage every 3 min. Our system is also equipped with environmental sensor arrays (one per cabinet) for real time assessments of ambient lighting and temperature during the recording, optional running wheels that can be added to the cages to provide greater running opportunities (and quantification of running behavior), and animals (25–250 g) can also be instrumented with a telemetric transducer for continuous recording of core temperature, gross motor activity, and heart rate (G2-HR E-mitter; Starr Life Sciences Corp, Oakmont, PA) upon request.

We recommend weighing food and drink hoppers before and after a Promethion recording session to ensure accuracy of the automated intake data sets. Issues arising from computer/electrical problems or accuracy of the measurements are infrequent but have been noted. While this does not provide granular data and validation of meal pattern analyses, it can still yield valuable information regarding overall food/water intakes if issues were to arise during data collection. Additionally, calibration of the gas analyzers, mass monitors, beam breaks as well as validations of the mass flow regulators *via* a high precision flow meter (MFM 0-5SLPM; Alicat; Tucson, AZ), and leak testing of the system are recommended prior to commencing an experiment to ensure rigor and reproducibility between experiments.

A typical workflow for the use of the MCW CRMPC Promethion system is to perform TD-NMR on the animals on day 0 (typically a Monday morning), immediately before animals are placed into the system. Animals then remain in the system continuously for ≈96 h and again undergo TD-NMR upon exiting the system (typically a Friday morning/afternoon). Investigators may choose to acclimate their animals to the cages (or an identical set of cages) for a few days before the initiation of the study, or more simply, to exclude data from the first 24–48 h of the 96 h experiment.

It is worthwhile to note that some strains of rodents are recalcitrant to study in multiplexed phenotyping systems. We strongly suggest that animals must be observed daily (at a minimum) to confirm food and water intake is occurring, especially when new strains of animals are introduced to these systems. In our experience with genetically diverse strains of rats, for example, we have observed (*unpublished observations*) that some strains exhibit exceptional neophobia when placed into multiplexed systems and must be removed from the systems after just 1–2 days of study due to a complete stoppage of food and/or water intake. It is incumbent upon investigators to ensure both the welfare of their subject animals and the validity of resulting datasets by ensuring proper acclimation to these housing/phenotyping systems; the design and ambience of such living quarters are not inert variables to the rodents simply because we humans deem it so (Soto et al., 2019; Ziegler et al., 2022).

Data analyses following an experiment using multiplexed systems can be onerous for the uninitiated, due to the large size of the resulting dataset and the complexity of the statistical approaches that are appropriate for these types of data. One online tool available publicly is the CalR website,^1^ which allows users to rapidly perform first-pass analyses of their data (Mina et al., 2018). Beyond this semi-automated approach, we strongly recommend that end users carefully evaluate datasets by manually processing data and using quality-control procedures including graphical presentations and assessments of variance to identify technical errors from individual datapoints throughout any resulting dataset. Primary outcomes from multiplexed phenotyping systems include food/drink hopper masses, respiratory gasses, and photoelectric beam interruptions versus time stamps; from this matrix of primary data, an exceptionally long list of secondary endpoints are derived. Calculations of food and fluid intake over time, meal and drink bout patterns, aerobic metabolic rates in different states (i.e., active versus resting) and respiratory exchange ratios, physical activity, sleep, and other endpoints are then performed.

#### Advantages and Disadvantages

The advantages of multiplex metabolic phenotyping are many, and largely stem from the temporal resolution provided by this approach. For example, this approach enables analysis of patterns (feeding, drinking, ambulation, metabolism, etc.) and interactions between these many measured endpoints. Further, the multiplexed nature of this approach accelerates the pace of research, as a number of animals can be studied for an array of endpoints within just a few days. In addition, refinements in multiplexed system design most recently include cage designs that closely resemble standard “home” caging designs, which presumably reduces psychological and physiological stresses on the animal. The primary disadvantage of this approach is the cost, which is typically prohibitive for individual investigators and generally represents a major institutional investment.

### Unrestrained Whole-Body Plethysmography

Gas exchange and acid–base chemistry of the blood are inextricably linked to metabolic physiology, and hindbrain control of breathing is also closely related to hindbrain mechanisms governing autonomic control of cardiovascular functions. Thus, our team also has a specific interest in quantitating ventilatory function in rodents.

Unrestrained whole-body plethysmography using the barometric method is a technique to measure breathing frequency and estimates of tidal volume and therefore, minute ventilation. Initially described by Drorbaugh and Fenn (1955), this method measures pressure deflections caused by inspiration and expiration. Within an enclosed chamber, with constant air flow (in and out) and chamber temperature, while an animal inspires, air entering the animal is heated and humidified causing volume expansion which increases the pressure within the chamber. The reverse occurs upon expiration. Measuring such pressure deflections provides a continuous waveform of inspiration and expiration for subsequent analyses and quantifications of breathing frequency and tidal volume. We, and many others, have adapted this method to measure breathing in a variety of species, including conscious mice (Hodges et al., 2008; Hodges and Richerson, 2008) and rats (Mouradian et al., 2012, 2017, 2019) of all ages.

**Figure 12:**
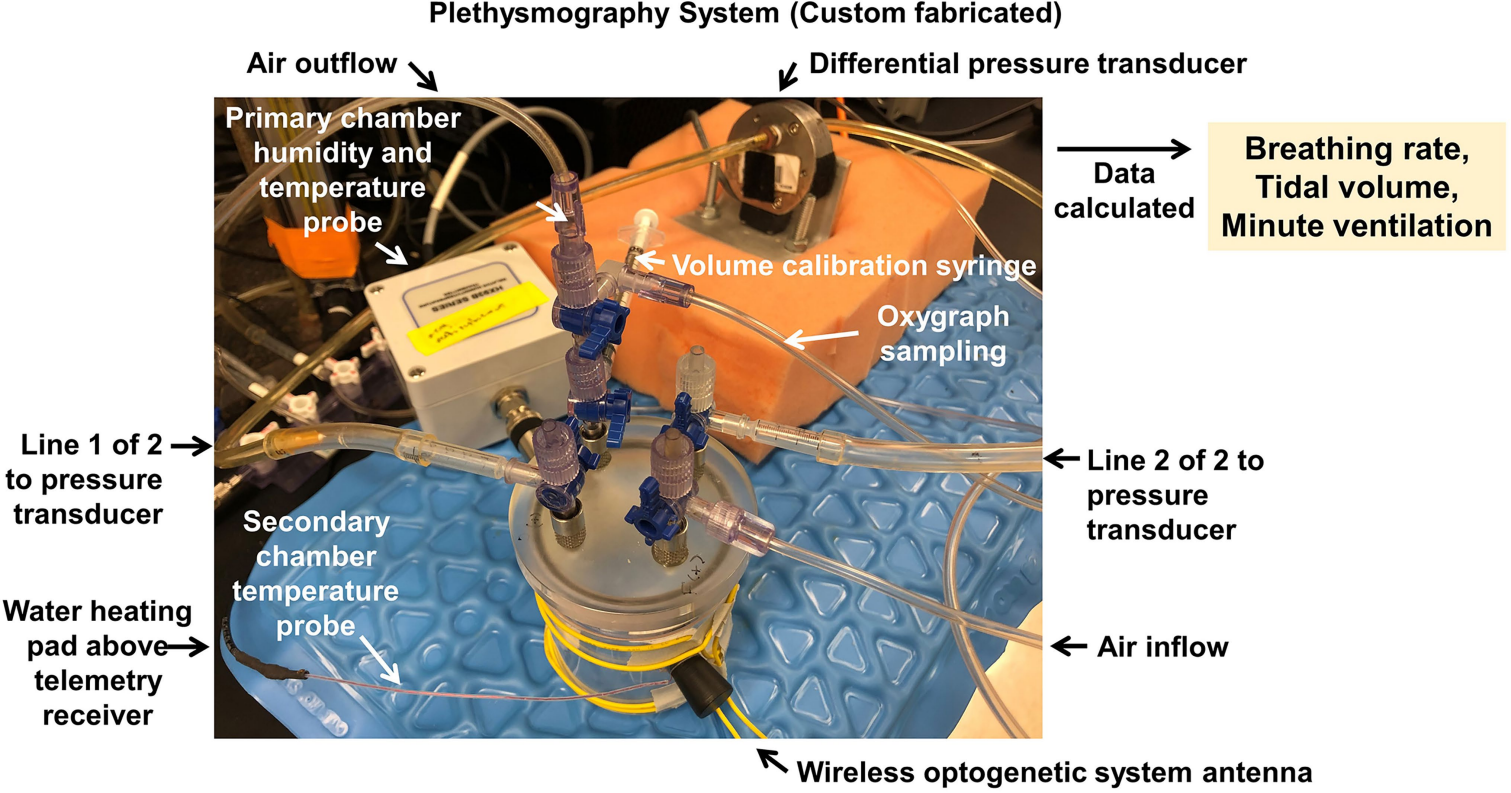
Plethysmography system. Custom built 200 ml mouse plethysmograph for the measurement of ventilatory functions during control and challenge (e.g., hypoxia or hypercapnia) conditions. Chamber is placed above a radiotelemetric receiver antenna to enable simultaneous measurements of blood pressure, electrical potential (e.g., electrocardiogram), core temperature, or other endpoints *via* previously implanted probes. Chamber is also interfaced with a wireless optogenetic system antenna assembly, to permit simultaneous optogenetic activation/inhibition of cellular functions *via* previously implanted wireless LED-based stimulators.

Briefly, in mice (all ages) and rats (<22 days old), animals are placed inside a custom 200 ml plethysmograph or 10 L plethysmograph (Figure 12). The gas inflow and outflow rates are balanced (150 ml/min) or slightly favoring more outflow to prevent accumulation of CO_2_. Varying gases are perfused into the chamber using the gas manifold connected to the gas inflow port. The temperature of the chamber is heated to assure 28–30°C within the chamber using a temperature-controlled heat source. Pressure deflections, measured in volts generated by inspiration and expiration, are measured using a differential pressure transducer (Validyne). Temperature and relative humidity are measured continuously (Omega). Analog signals are connected to an A/D convertor and recorded using a data acquisition system (Windaq or PowerLab) at 200 Hz sampling rate. Rectal temperatures from each animal are obtained using a T-type rectal thermocouple after each experiment.

A general workflow consists of each study beginning with 20–40 min of room air breathing, allowing sufficient time for the animal to calm under room air conditions. Then, for 10–20 min, either hypoxic (12%O_2_, 0%CO_2_, and 88% N_2_) or hypercapnic (21%O_2_, 7%CO_2_, and 71%N_2_) gases are infused into the chamber to measure ventilatory sensitivities to oxygen and carbon dioxide. Periods of calm breathing in the last half of each breathing condition are spliced together for subsequent quantification of breathing frequency and tidal volume.

Data collected from each study are analyzed offline in LabChart (ADInstruments). Each peak in the ventilatory waveforms are identified using the “cyclic measurement” option. Then, the peak voltage deflection from start to end of each breath is identified and measured using the “height measurement” option. Volts are converted into volume (milliliters) using a volume calibration and are corrected for animal and chamber temperature, relative humidity, and ambient barometric pressure to estimate tidal volume per breath. (Drorbaugh and Fenn, 1955) The rate of breathing frequency is quantified using the “cyclic measurements” option in LabChart. Minute ventilation (VE; ml/min) is calculated as the product between breathing frequency (F; breaths/min) and tidal volume (VT; ml/breath). All ventilation waveforms selected for quantification are devoid of breathing (apneas, sighs, and sniffs) and movement artifacts. Ventilation measured during either challenge condition (hypoxia or hypercapnia) is divided by room air breathing and multiplied by 100 percent to assess respiratory system sensitivity to oxygen and carbon dioxide.

#### Advantages and Disadvantages

Whole-body plethysmography provides minimally invasive assessments of breathing without the need for anesthesia, and this approach can be multiplexed with other methods. Our system, for example, is additionally multiplexed with antennas for radiotelemetric measures of blood pressure or other endpoints, and with stimulators for optogenetic manipulations. Disadvantages of this approach include the inability to assess lung resistance and compliance, for example.

### Radiotelemetric Blood Pressure Trasnsducers

**Figure 13:**
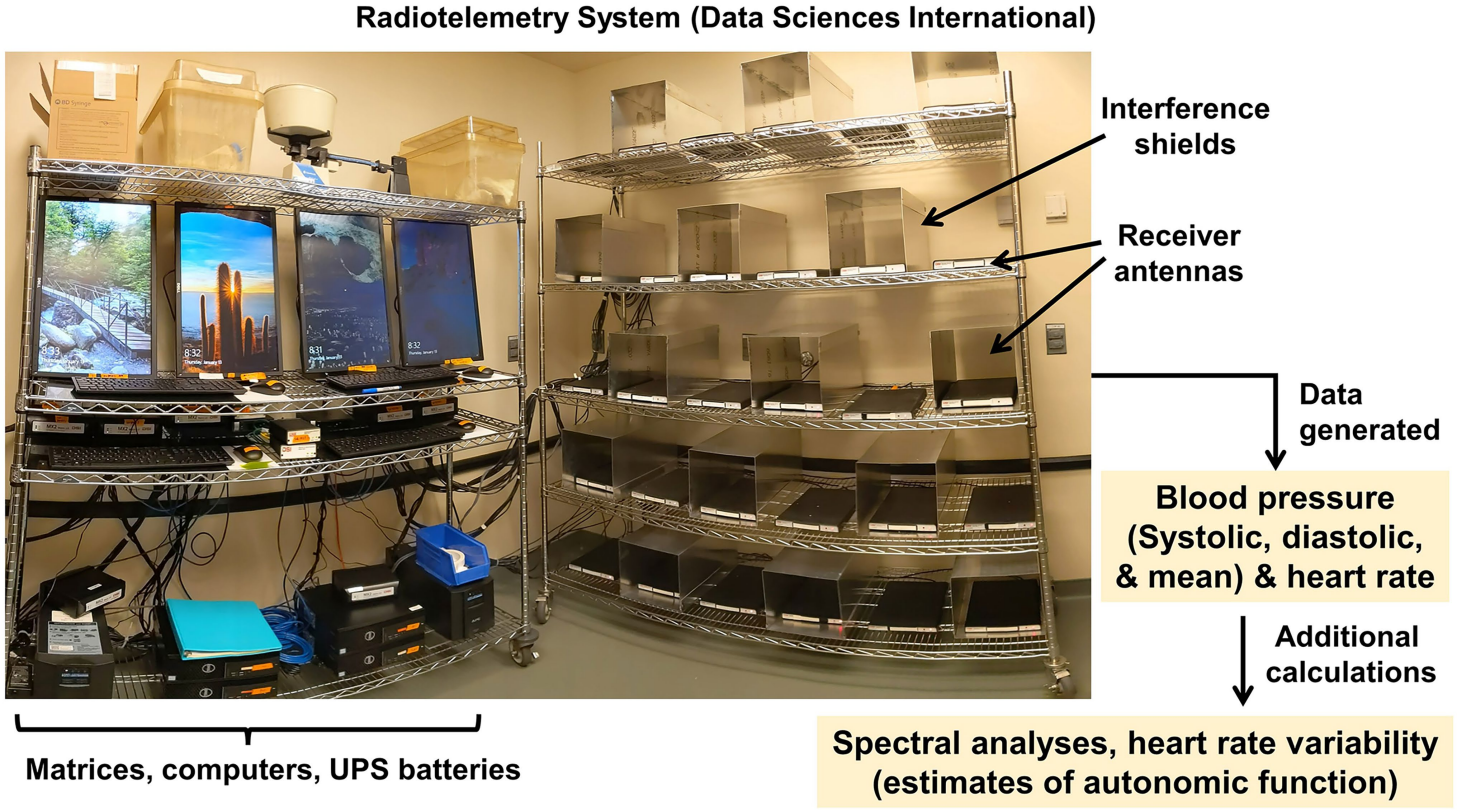
Radiotelemetry system. Data Sciences International radiotelemetric transducer system permits acquisition of data from a wide array of implanted probe types; most commonly, we use these systems for recording blood pressure and heart rate data throughout the day/night cycle.

Radiotelemetry is considered a gold standard method for the direct measurement of blood pressure in rodent models, as it permits 24 h recording of the full pressure waveform (Kurtz et al., 2005). An important advantage of radiotelemetric recordings is that animals are not subjected to handling, restraint, or thermal stresses, any of which may introduce confounding effects. We and our team have extensively used DSI telemetry systems to acquire blood pressure data for several decades to great effect (Jo et al., 2015; Muta et al., 2016; Shinohara et al., 2016; Sandgren et al., 2018; Nakagawa et al., 2021; Zanaty et al., 2021; Figure 13). The radiotelemetry array currently in use by our team includes 60 independent receiver antennas, permitting data collection from up to 60 animals simultaneously. Given the high demand, these receiver antennas are distributed across 4 independent matrices and workstations, which can be monitored remotely, so that researchers can start new protocols without interrupting other ongoing experiments. For blood pressure measurements, we typically use the TA11PA-C10, HD-X10, HD-S10, or similar implantable radiotelemetric probe (DSI, New Brighton, MN), which is surgically introduced in the left carotid artery by a highly trained surgeon. Once recovered from the implantation surgery, the animal is housed individually in a static cage placed on top of the telemetry receiver antenna, with free access to water and pelleted diet.

Blood pressure and heart rate data are acquired after 10 days of post-surgical recovery. Typically, blood pressure and heart rate data are recorded at 500 Hz for 10 s every 5 min for 24 h, and averaged daily, hourly, and during the light (5 AM–7 PM) and dark phase (7 PM–5 AM). Data are collected at a higher rate when beat-by-beat heart rate values are needed for heart rate variability analyses. Such analyses can provide an estimate of autonomic activity without any additional intervention, though any suggestive result should be confirmed by pharmacological or surgical methods.

For example, after measuring baseline parameters, our studies typically include different acute or chronic challenges. Acute challenges may include intracerebroventricular infusions of peptides or pharmacological agents, intraperitoneal injections of beta-adrenergic blockers, or muscarinic receptor antagonists to assess sympathetic or parasympathetic nervous activities, respectively, or acute exposures to hypercapnia or hypoxia (as described above) when plethysmography+radiotelemetry studies are combined. Chronic challenges might include subcutaneous delivery of deoxycorticosterone salts plus excess sodium in drink solutions or food sources (i.e., DOCA-salt treatment), subcutaneous or intracerebroventricular infusions of angiotensin II *via* osmotic minipump, or changes in the diet (i.e., high-fat or high-sodium diets). All data are collected and analyzed using the Ponemah software platform (DSI New Brighton, MN).

#### Advantages and Disadvantages

Radiotelemetric approaches are considered by many to be the best approach for assessing blood pressure in rodents, as these methods directly measure pressure in the cannulated vessel, and provide 24 h access to such assessments in animals that are unrestrained and living in a standard “home” cage. The primary disadvantages of this approach include the exceptionally high cost of telemetric transducers, the short battery life of the transducers, and the requirement to surgically implant the transducer which requires anesthesia and typically involves distal occlusion of the cannulated vessel.

### Direct Calorimetry and Combined Calorimetry

From a physical or chemical perspective, metabolic rate is the rate of heat production from biochemical reactions. As a result, metabolic rate is reported in units of energy (or heat) flux, such as kcal/h. The fundamental principle of *direct* calorimetry is as the name implies: the direct measurement of heat flux. This is as opposed to *indirect* calorimetry, in which heat flux is indirectly estimated through the measurement of other parameters, such as oxygen & carbon dioxide exchange in a gas respirometer. When the rate of heat accumulation in the body is at steady state (i.e., the core body temperature is stable), heat dissipation as measured in a direct calorimeter is equivalent to heat production (i.e., metabolic rate). Conceptually:


[Heatproduction]=[Heatdissipation]+[Heataccumulation]

Heat is transferred from an organism to its environment *via* conduction, convection, radiation, and phase changes (e.g., vaporization of water in the respiratory tract). These means of heat transfer ultimately result in heat transfer from the rodent to the air moving through the calorimetry chamber, plus heat flux through the walls of the chamber. Additionally, bodily retention of heat manifests as changes in core temperature. Expanding this to measured terms:


$$\begin{array}{l} \left[Heatdissipation\right]=\left[Heatfluxthroughcalorimeterwalls\right]+\\ \left[Changesinenthalpyof\;air\right]+\left[\begin{array}{l} Changesincorebody\\ temperature \end{array}\right] \end{array}$$

**Figure 14:**
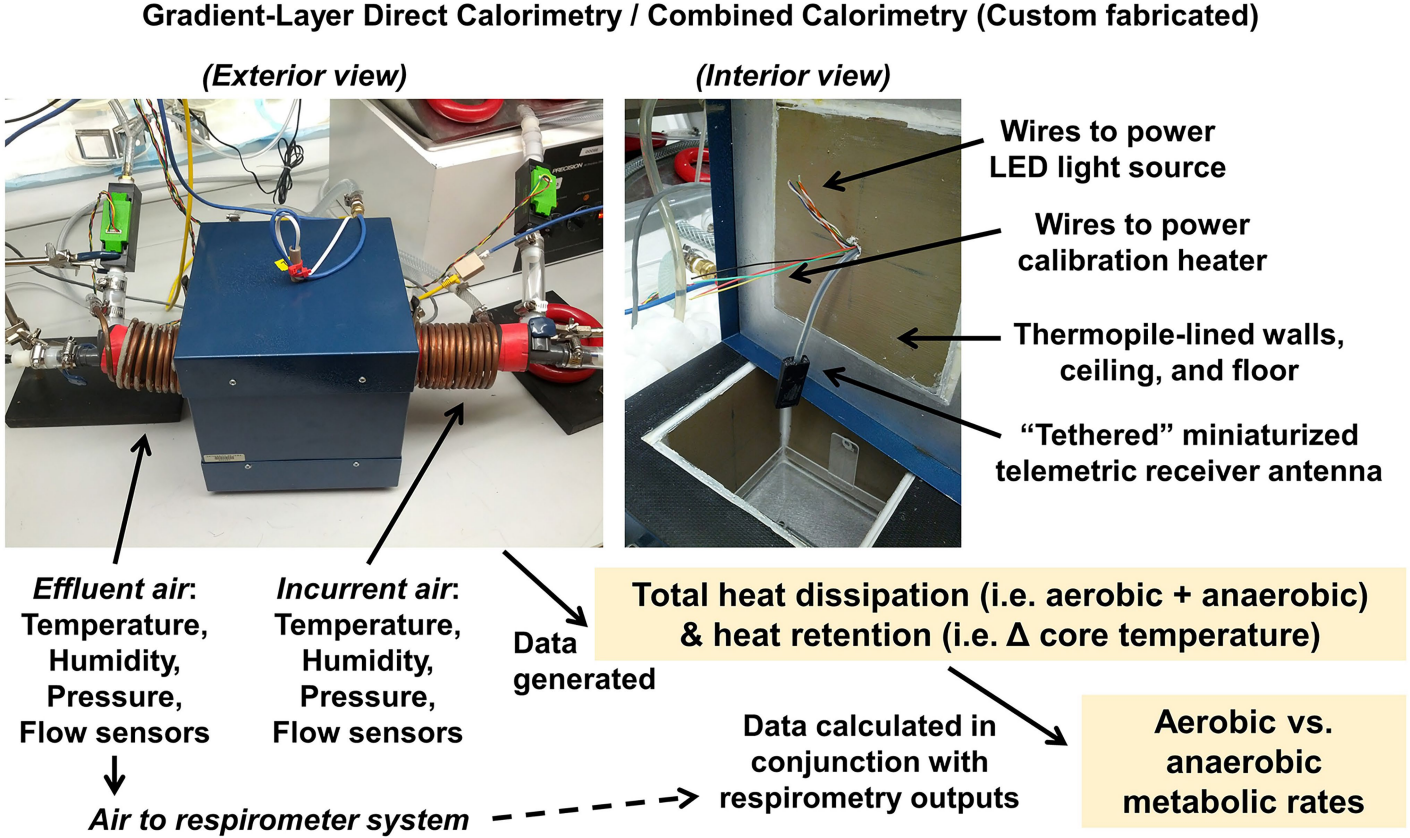
Gradient-layer direct calorimeter. Custom-fabricated gradient-layer direct calorimeter system partially manufactured by Heinz Poppendiek, PhD (Geoscience, Ltd). System is additionally interfaced with an O_2_/CO_2_ gas respirometry system to permit simultaneous measurement of gas exchange.

As previously described in great detail (Burnett and Grobe, 2013, 2014; Grobe, 2017), the direct calorimeter chamber currently in use by our team for mice and very small rats is a gradient-style calorimeter in which a cube (interior dimensions: 10x10x10 cm) of thermopiles is attached on the inside of thermally controlled metal jacket (Figure 14). Influent air is conditioned to provide a near constant enthalpy by controlling the humidity through cooling of saturated air to a target dew point (typically 10°C), then warmed to thermoneutrality (i.e., 30°C for a C57BL/6 J mouse), which is identical in temperature to the thermally controlled jacket of the box. As heat fluxes through the thermopile-lined walls to the heat sink, a voltage is produced that is linearly proportional to the heat flux. Additionally, the change in the enthalpy of the air is calculated to account for heat transfer to the influent air as it is breathed and leaves the box. Specially constructed sensors measure the pressure, humidity, temperature, and mass flow on both the influent air and effluent air to yield the difference (at STP) in enthalpy contributed by the rodent. Data from all sensors are collected into LabView (National Instruments) for further analyses. Simultaneously, a custom-fabricated, miniaturized antenna (Data Sciences International) is located inside the calorimetry chamber to permit continuous recording of core body temperature of the rodents from previously implanted radiotelemetric core temperature probes.

Finally, to form a “combined calorimeter,” this direct calorimeter is placed inline with the respirometer (i.e., indirect calorimeter) system described above. The gas composition of the influent and effluent air streams is measured continuously before, during, and after the experiment, which closely resembles the timeframe of respirometry experiments as described above. Aerobic heat is then estimated as described above, and the difference between total measured metabolic rate (from direct calorimetry) and estimated aerobic metabolic rate (from respirometry) is considered the “non-aerobic” (or “anaerobic”) metabolic rate.


$$\begin{array}{l} \left[Totalheatproduction\right]=\left[Aerobicheatproduction\right]+\\ \;\;\;\;\;\;\;\;\;\;\;\;\;\;\;\;\;\;\;\;\;\;\;\;\;\;\;\;\;\;\;\;\;\;\;\;\;\;\;\;\;\;\;\;\;\left[Anaerobicheatproduction\right] \end{array}$$

In other words, we hypothesize:


$$\begin{array}{l} \left[Directcalorimetryresult\right]=\left[Respirometryresult\right]+\\ \;\;\;\;\;\;\;\;\;\;\;\;\;\;\;\;\;\;\;\;\;\;\;\;\;\;\;\;\;\;\;\;\;\;\;\;\;\;\;\;\;\;\;\;\;\;\;\;\;\;\;\left[Anaerobicmetabolism\right] \end{array}$$

On the day of the experiment, the rodent is removed from their home cage and transferred into the direct calorimeter box. The box, which is heavily insulated and surrounded on all sides by a tightly regulated water jacket, includes a small white LED on the interior (which generates a small but specifiable amount of heat) that can be enabled to maintain the light cycle. The rodent is allowed to explore the interior of the chamber and, ultimately, achieves a resting state in the 4–6 h fasting time frame. Alternatively, we have also used the system with mice under anesthesia, which permits analyses before/after rapid surgical or pharmacological manipulations and greatly accelerates the testing timeframe (Riedl et al., 2021). After the instantaneous data appear to have been stable for an acceptable time, the rodent is removed from the box and returned to their home cage to eat and drink. The total heat and aerobic heat are calculated from the data recorded during the “rest” phase and time-averaged over this period.

#### Advantages and Disadvantages

Direct calorimetry offers technical superiority over other methods of assessing metabolic rate. Relative to gas respirometry, direct calorimetry provides similar temporal resolution which permits dissociation of active versus resting metabolic rates, but direct calorimetry is not subject to the long list of unlikely assumptions that plague respirometry and it uniquely captures additional forms of heat production including protein and anaerobic metabolism. The single greatest disadvantage of this approach is a lack of commercial availability (i.e., investigators must build and validate their own custom systems). Additionally, because direct calorimetry includes the measurement of heat loss through evaporation, studies using this approach are complicated by the presence of water sources or urine in the chamber. Thus, design hurdles must be overcome before direct calorimetry will make its way into multiplexed phenotyping system designs.

### Anaerobic Metabolism by Multiplexed Systems?

In addition to the many other endpoints determined by multiplexed phenotyping systems, our team has forwarded the concept that metrics of anaerobic metabolism may also be estimated from the vast datasets produced by multiplexed systems (Soto et al., 2019).

As a general concept, total energy balance can be expressed as follows:


[EnergyAbsorbed]=[Growth]+[EnergyExpenditure]

Expanding these generalized terms into slightly more specific mechanisms, the equation becomes:


$$\begin{array}{l} \left[CaloriesConsumed\right]\times \left[DigestiveEfficiency\right]=\left[Growth\right]+\\ \left[AerobicExpenditure\right]+\left[UnaccountedCalories\right] \end{array}$$

Using TD-NMR to determine body composition before and after a ≈ 96 h analysis period within a Promethion multiplexed phenotyping system, we can determine the change in fat and fat-free masses during that timeframe. If we estimate that fat mass is worth 9 kcal/g and fat-free mass (FFM) is worth 4 kcal/g, this yields a kcal equivalent for the growth variable:


$$\left[Growth\right]=9\times \left[\Delta \;fat\;mass\right]+4\times \left[\Delta \;FFM\right]$$

Caloric consumption is simply determined as the accumulated food intake by the animal across the entire ≈96 h analysis period. Similarly, aerobic expenditure is estimated by the respirometric gas exchange across the entire ≈96 h analysis period.

Finally, digestive efficiency can be determined by bomb calorimetry by collecting the feces produced in the Promethion system over the 96 h period, or using feces collected from the same animals when housed in home cages or in metabolic cages during the week preceding or following the 96 h Promethion run, or using feces collected from a parallel cohort of animals.

By inserting all of these values into the above equation, we can solve for the remaining variable (Unaccounted Calories). Previously, we have demonstrated that this variable can account for an enormous fraction of total energy turnover in mice and that the effect is greatly dependent upon diet and sex. For example, in male mice fed a 60% ultra-high-fat diet for 5 weeks, there are essentially 0% unaccounted calories; in contrast, in female mice fed the same diet for the same timeframe, approximately 50% of consumed calories are unaccounted (Soto et al., 2019).

To date, we have performed a small number of preliminary studies that include parallel experimental paradigms (i.e., the same sex, timeline, diets, and experimental interventions) studied using both combined calorimetry and this novel multiplexed phenotyping approach. Intriguingly, these studies support the concept that “unaccounted calories” may indeed represent a metric of anaerobic metabolism. Ongoing experiments including manipulation of the gut microbiota to test this concept more directly are required. If supported, then the establishment of “unaccounted calories” as a reliable metric of anaerobic metabolism *in vivo* would provide an outstanding, high-throughput tool to study the role of the gut microbiota in whole-body energetic flux.

#### Advantages and Disadvantages

This approach is not yet validated, and many careful studies are needed to get to that point. If validated, however, this “subtractive’ approach to estimate anaerobic metabolism will offer enormous advantages including the ability to assess this form of energy flux in a non-invasive, high-throughput format. The development and validation of such an approach would revolutionize studies of the contribution of the gut microbiota to whole-body energy flux.

## Limitations

The objective of the MCW CRMPC is to provide low-barrier access to an array of cutting-edge technologies and expert guidance for studying metabolic function in rodents. The various approaches outlined here can provide a wealth of information regarding energetic flux and cardiometabolic functions, but it would be negligent to fail to note that other approaches and technologies are not provided (at this time) by the CRMPC which could also provide additional insights. For example, for investigators interested in glycemic control biology, various methods including glucose, insulin, and other tolerance tests provide initial insights. Such studies are often followed by glycemic or insulin clamping studies to confirm and extend those studies. Isotope dilution studies are another robust approach to tracking metabolic processes, which offer the unique advantage of allowing animals to be returned to their normal habitat during such studies. Radiolabeled tracer studies also provide investigators the ability to track substrate uptake by individual tissues, which can be leveraged to better understand the responsible organs when changes in whole-body energy expenditure are observed. Finally, a wealth of approaches are encompassed within the umbrella of “-omics” methods (including, but clearly not limited to: genomics, transcriptomics, translatomics, proteomics, and metabolomics) that can be utilized to dissect the influence of genes, environment, and other factors upon energy flux.

## Summary and Conclusion

In summary, the MCW CRMPC and collaborating laboratories have developed an outstanding array of high-throughput, high-resolution, and complementary tools to comprehensively evaluate energy, fluid, cardiovascular, ventilatory, and autonomic functions in mouse and rat models. This streamlined toolbox is well suited to support an array of cutting-edge studies across disciplines and research foci. In addition, we continue to not only provide access to expert guidance and technical support for current cutting-edge technologies such as TD-NMR and multiplexed systems, but we also strive to stay at the forefront of innovation through ongoing development of the newest technologies such as direct and combined calorimetry-type systems, and through the optimization of approaches to study and manipulate the gut microbiota. Through a combination of institutional, departmental, and laboratory financial & technical support, plus the organic organization and research specialty of the collaborative group, the team and facility are positioned to make significant contributions to cardiometabolic research efforts within the MCW community. As our facility grows, we look forward to continue developing workflows and mechanisms to support investigations by researchers outside of MCW.

## Data Availability Statement

The original contributions presented in the study are included in the article/supplementary material, further inquiries can be directed to the corresponding author.

## Ethics Statement

The animal study was reviewed and approved by Medical College of Wisconsin Institutional Animal Care and Use Committee.

## Author Contributions

JR, PN, GM, CG, FS, CB, and JG drafted the portions of the manuscript. JR, AK, JK, JS, MH, CS, and JG edited the manuscript. All authors contributed to the article and approved the submitted version.

## Funding

This work and the authors were supported by grants from the NIH (HL084207, HL134850, HL144807, HL153101, ES005605, HL152495, OD024617, AI108255, and HL122358), the American Heart Association (18EIA33890055 and 20CDA3510121), the Children’s Research Institute (CRI22700), the MCW CTSI (UL1TR001436), the Advancing a Healthier Wisconsin Endowment, and the MCW Department of Physiology.

## Conflict of Interest

The authors declare that the research was conducted in the absence of any commercial or financial relationships that could be construed as a potential conflict of interest.

## Publisher’s Note

All claims expressed in this article are solely those of the authors and do not necessarily represent those of their affiliated organizations, or those of the publisher, the editors and the reviewers. Any product that may be evaluated in this article, or claim that may be made by its manufacturer, is not guaranteed or endorsed by the publisher.

## Footnotes

^1^ www.calrapp.org
